# Causal association of circulating cytokines with the risk of lung cancer: a Mendelian randomization study

**DOI:** 10.3389/fonc.2024.1373380

**Published:** 2024-06-18

**Authors:** Dachen Luo, Zonglian Gong, Qingyuan Zhan, Shan Lin

**Affiliations:** ^1^ Department of Respiratory and Critical Care Medicine, Affiliated Hospital of North Sichuan Medical College, Nanchong, China; ^2^ Department of Pulmonary and Critical Care Medicine, Center of Respiratory Medicine, National Center for Respiratory Medicine, China-Japan Friendship Hospital, Beijing, China

**Keywords:** cytokines, lung cancer, Mendelian randomization, causality, genome-wide association study

## Abstract

**Background:**

Lung cancer is the deadliest and most prevalent malignancy worldwide. While smoking is an established cause, evidence to identify other causal factors remains lacking. Current research indicates chronic inflammation is involved in tumorigenesis and cancer development, though the specific mechanisms underlying the role of inflammatory cytokines in lung cancer pathogenesis remain unclear. This study implemented Mendelian randomization (MR) analysis to investigate the causal effects of circulating cytokines on lung cancer development.

**Methods:**

We performed a two-sample MR analysis in Europeans utilizing publicly available genome-wide association study summary statistics. Single nucleotide polymorphisms significantly associated with cytokine were selected as genetic instrumental variables.

**Results:**

Genetically predicted levels of the chemokine interleukin-18 (IL-18) (OR = 0.942, 95% CI: 0.897–0.990, P = 0.018) exerted significant negative causal effects on overall lung cancer risk in this analysis. Examining specific histologic subtypes revealed further evidence of genetic associations. Stem cell factor (SCF) (OR = 1.150, 95% CI: 1.021–1.296, P = 0.021) and interleukin-1beta (IL-1β) (OR = 1.152, 95% CI: 1.003–1.325, P = 0.046) were positively associated with lung adenocarcinoma risk, though no inflammatory factors showed causal links to squamous cell lung cancer risk. Stratified by smoking status, interferon gamma-induced protein 10 (IP-10) (OR = 0.861, 95% CI: 0.781–0.950, P = 0.003) was inversely associated while IL-1β (OR = 1.190, 95% CI: 1.023–1.384, P = 0.024) was positively associated with lung cancer risk in ever smokers. Among never smokers, a positive association was observed between lung cancer risk and SCF (OR = 1.474, 95% CI: 1.105–1.964, P = 0.008). Importantly, these causal inferences remained robust across multiple complementary MR approaches, including MR-Egger, weighted median, weighted mode and simple mode regressions. Sensitivity analyses also excluded potential bias stemming from pleiotropy.

**Conclusion:**

This MR study found preliminary evidence that genetically predicted levels of four inflammatory cytokines—SCF, IL-1β, IL-18, and IP-10—may causally influence lung cancer risk in an overall and subtype-specific manner, as well as stratified by smoking status. Identifying these cytokine pathways that may promote lung carcinogenesis represents potential new targets for the prevention, early detection, and treatment of this deadly malignancy.

## Introduction

As the deadliest cancer worldwide, lung cancer was responsible for 1,817,000 deaths in 2022, accounting for 18.7% of all cancer-related fatalities (1). Due to typically late-stage diagnosis once symptoms arise, the 5-year survival rate for lung cancer patients is dismally low at around 20% (2). Smoking is a well-known predominant risk factor, yet evidence to elucidate other potential causes remains scarce (3, 4). Identifying additional contributory risk factors to promote early detection and treatment thus represents a critical need in combating this devastating disease.

Over the past two decades, a sizable body of evidence has firmly established chronic infection and inflammation as key promoters of carcinogenesis (5–8). The inflammatory tumor microenvironment, comprised of leukocytes releasing cytokines, chemokines, reactive oxygen species and other cytotoxic mediators, can drive tumor progression through processes such as genotoxicity, aberrant tissue repair, heightened cellular proliferation, invasion and metastasis (5, 9). Critical transcription factors including STAT3 and NF-κB have been implicated in inflammation-fueled carcinogenesis (10, 11). Tumors can further manipulate the inflammatory milieu to suppress anti-tumor T cell responses (12). However, despite substantial links between cytokines and cancer, the precise underlying mechanisms, particularly in lung cancer, remain to be fully deciphered.

Mendelian randomization (MR) analysis utilizes genetic variation as an instrumental variable to infer the potential causal relationship between modifiable exposures and disease outcomes (13). As genetic variation is randomly assigned and not susceptible to reverse causation, this ingenious approach circumvents confounding and reverse causation bias that plague traditional observational studies (14). In this study, we implemented MR to investigate the causal role of circulating cytokines in lung cancer pathogenesis. Using genetic instruments as surrogates for cytokine levels, we evaluated whether inflammation actively drives lung cancer development or simply represents an epiphenomenon. Elucidating these causal pathways will provide novel mechanistic insights and could reveal previously unrecognized therapeutic targets for this pernicious lung disease.

## Method

### Study design

In this two-sample MR analysis, we utilized single nucleotide polymorphisms (SNPs) as instrumental variables (IVs). To ensure validity, SNPs were chosen based on three critical assumptions: (1) significant associations with exposure factors, satisfying relevance; (2) effects on outcomes solely via exposures, not through alternative pathways, fulfilling exclusivity; and (3) independence from confounding factors (15).

### Data resource

SNPs associated with circulating cytokines were obtained from the latest genome-wide association studies (GWAS), as listed in **Supplementary Table 1**. Summary data from a large-scale cytokine GWAS meta-analysis were used to generate genetic instruments for cytokines (16). This meta-analysis measured cytokine levels in plasma and blood samples from 8,293 Finnish individuals across three population-based cohorts.

To investigate causal effects of circulating cytokines on lung cancer risk, we obtained lung cancer GWAS summary statistics from the IEU open database. Lung cancer data were obtained from the International Lung Cancer Consortium (ILCCO) (https://ilcco.iarc.fr/), including 29,836 cases and 55,586 controls (17). The study also provided associations between instrumental SNPs and different histologic subtypes of lung cancer, including lung adenocarcinoma (case: 11,245, control: 54,619), squamous cell carcinoma (case: 7,704, control: 54,763), and small cell lung cancer (case: 2,791, control: 20,580). Subgroup analyses were performed according to smoking status, including smokers (23,223 cases and 16,964 controls) and never-smokers (2,355 cases and 7,504 controls), and all populations were restricted to European ethnic groups.

### SNPs selection

To identify valid SNPs for MR, we implemented several filtering steps: First, we selected independent SNPs strongly associated with different cytokine levels (*P <*5×10^−6^) to maximize instrument availability (18, 19). Second, we used clumping to prune correlated SNPs in linkage disequilibrium (r^2^<0.001, 10,000 kb) and avoid biased results. Third, we excluded pleiotropic SNPs associated with potential confounders including smoking, diabetes and anxiety using PhenoScanner (20). Fourth, we retained only concordant SNPs between exposure and outcome datasets as valid instruments. Finally, we excluded weak instruments with F-statistics <10 (calculated as F = R^2^×(N-2)/1-R^2^) to minimize bias (21).

### Statistical analysis

After SNP filtering, our primary MR analysis utilized inverse-variance weighted (IVW) estimation to evaluate the overall causal effects, given its accuracy when all instruments are valid (22). Complementary approaches including weighted median, MR Egger, weighted mode and simple mode were also implemented (23, 24). To probe potential horizontal pleiotropy, we performed MR Egger regression and Mendelian randomization pleiotropy residual sum and outlier (MR-PRESSO) testing (25, 26). Heterogeneity was assessed via Cochran’s Q and MR Egger regression, and result robustness was verified through leave-one-out analysis. Furthermore, we conducted Steiger testing to evaluate possible reverse causation (27).

To correct for multiple hypothesis testing, we applied Bonferroni correction and set statistical significance at P < 0.0012 based on the number of cytokines analyzed. P-values ranging from 0.0012 to 0.05 were considered suggestive evidence for potential causality (28, 29). All Mendelian randomization analyses were implemented in R utilizing the TwoSampleMR and MR-PRESSO packages. A P-value below 0.05 was deemed statistically significant.

## Results

### Causal effects of circulating cytokines on lung cancer and different subtypes

**Figure 1:**
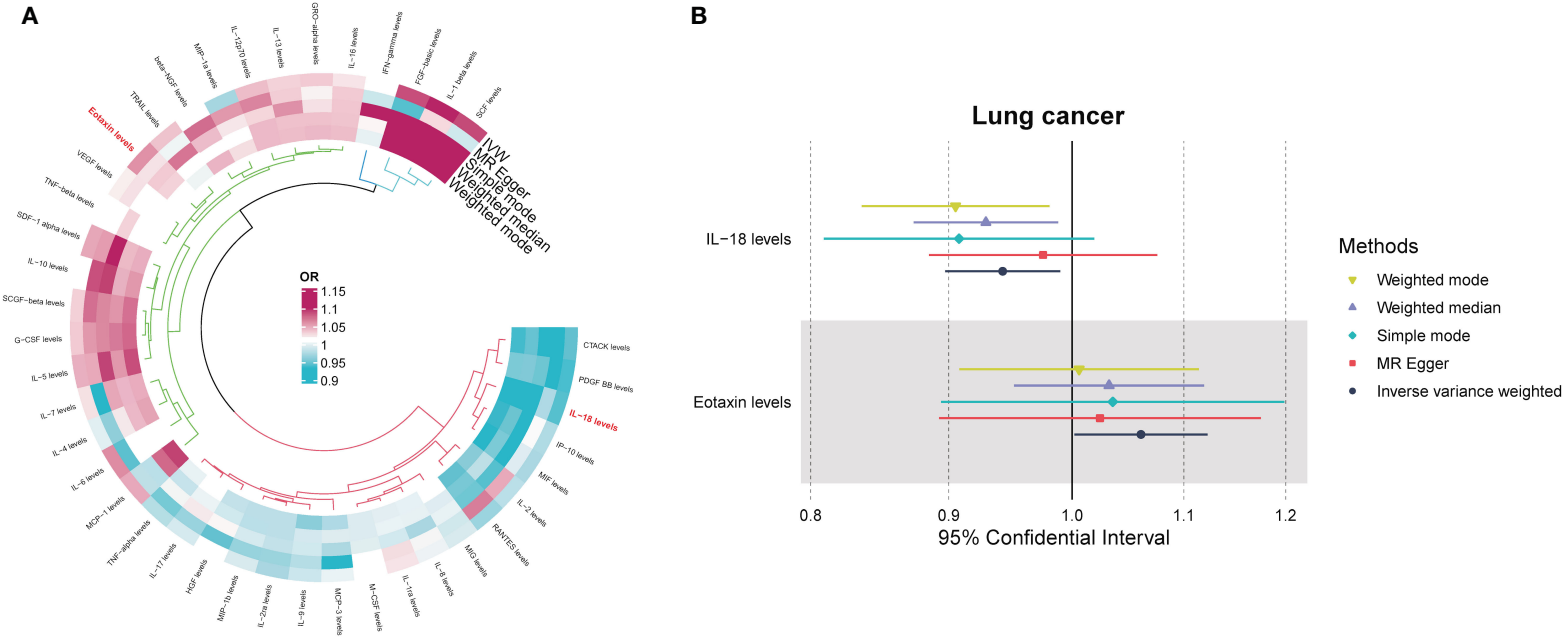
**(A)** Odds ratio cyclic heatmap of the MR analysis results for circulating cytokines and risk of lung cancer. **(B)** Forest plot of results with statistical significance.

After implementing the described filtering procedures, 4–16 SNPs were retained as instruments for the circulating cytokines (**Supplementary Table 1**). High F-statistics confirmed all selected SNPs were robust instruments (all F values >10). We then leveraged these SNPs to infer causality between cytokines and lung cancer. MR estimates from the various analytical methods are visualized in **Figure 1** (**Supplementary Table S2**). Specifically, IVW analysis revealed interleukin-18 (IL-18) exerted significant negative causal effects on lung cancer risk (OR = 0.942, 95% CI: 0.897–0.990, P = 0.018), while eotaxin had significant positive causal effects (OR = 1.061, 95% CI: 1.002–1.123, P = 0.043) (**Figures 1A, B**). Eotaxin, however, has a statistical efficacy of only 67%, thus it remains to be verified. Consistent estimates were yielded by MR Egger and weighted median methods. Scatter plots visualized the effects for each method across datasets (**Supplementary Figure 1**).

**Figure 2:**
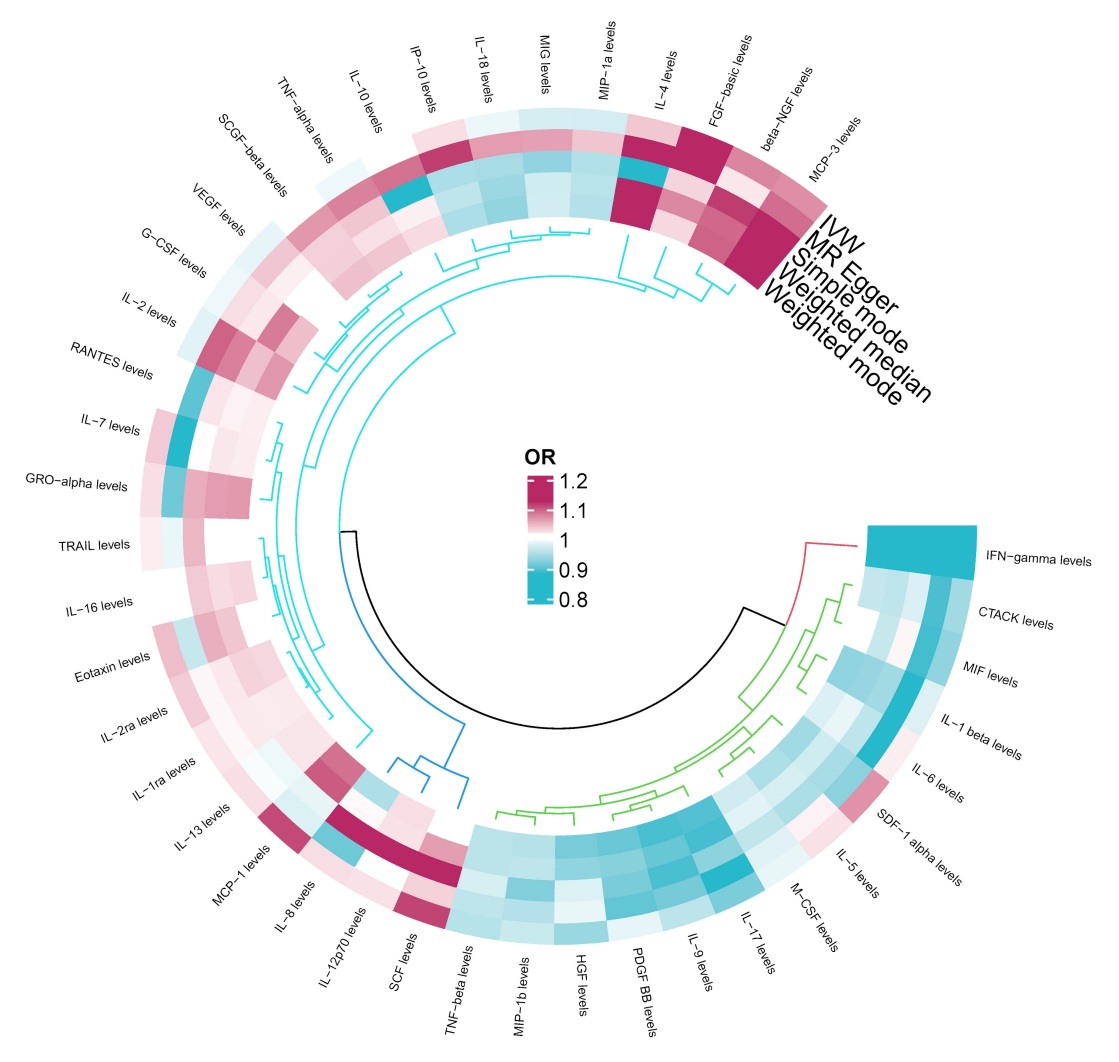
Odds ratio cyclic heatmap of the MR analysis results for circulating cytokines and risk of squamous cell lung cancer.

**Figure 3:**
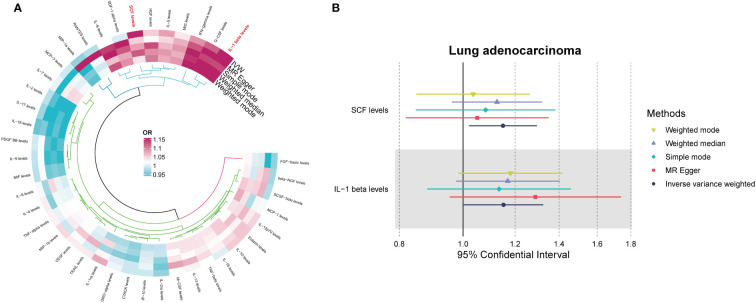
**(A)** Odds ratio value cyclic heatmap of the MR analysis results for circulating cytokines and risk of lung adenocarcinoma. **(B)** Forest plot of results with statistical significance.

**Figure 4:**
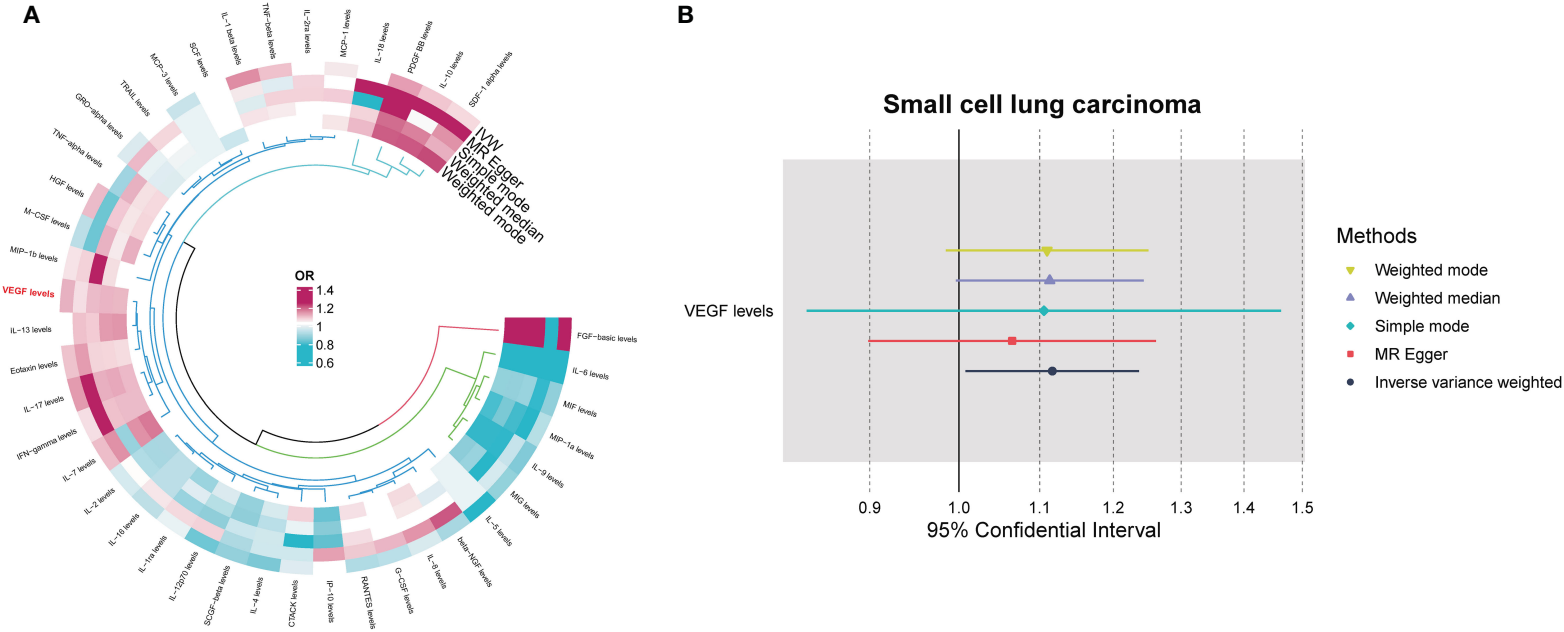
**(A)** Odds ratio value cyclic heatmap of the MR analysis results for circulating cytokines and risk of small cell lung carcinoma. **(B)** Forest plot of results with statistical significance.

Among the different histologic lung cancer types, we found further evidence of genetic associations (**Figures 2**-**4**). No inflammatory factor was found to be causally associated with the risk of squamous cell lung cancer (**Figure 2**) (**Supplementary Table S3**). Stem cell factor (SCF) (OR = 1.150, 95% CI: 1.021–1.296, P = 0.021) and interleukin-1beta (IL-1β) (OR = 1.152, 95% CI: 1.003–1.325, P = 0.046) were positively associated with the risk of lung adenocarcinoma (**Figures 3A, B**) (**Supplementary Table S4**). Vascular endothelial growth factor (VEGF) (OR = 1.117, 95% CI: 1.008–1.237, P = 0.035) was positively associated with the risk of small cell lung carcinoma (**Figure 4A, B**) (**Supplementary Table S5**), but the statistical efficacy of VEGF was limited to 21%, which makes the causal hypothesis unreliable. Scatter plots visualized the effects for each method across datasets (**Supplementary Figures 2**, **3**).

MR Egger regression and MR-PRESSO global testing showed no evidence of horizontal pleiotropy (**Table 1** and **Supplementary Tables 6**-**9**). Importantly, Cochran’s Q statistics and MR Egger regression revealed no significant heterogeneity among the individual SNP instruments (P > 0.05). MR-PRESSO global test p-values exceeding 0.05 for all cytokine-lung cancer associations, including different pathological types, further ruled out pleiotropy. Leave-one-out analysis confirmed the robustness of the causal estimates (**Supplementary Figures 4**-**6**).

**Table 1:** Primary results of MR analysis on squamous cell lung cancer.

	Method	Beta	SE	OR	95% CI	*P* -value	LOW	UP
CTACK levels	Inverse variance weighted	-0.059	0.048	0.942	0.858-1.035	0.214	0.858	1.035
beta-nerve growth factor levels	Inverse variance weighted	0.076	0.076	1.079	0.930-1.253	0.317	0.930	1.253
Vascular endothelial growth factor levels	Inverse variance weighted	-0.011	0.033	0.989	0.927-1.056	0.749	0.927	1.056
Macrophage Migration Inhibitory Factor levels	Inverse variance weighted	-0.070	0.063	0.932	0.824-1.055	0.265	0.824	1.055
TRAIL levels	Inverse variance weighted	0.018	0.033	1.018	0.955-1.086	0.580	0.955	1.086
Tumor necrosis factor beta levels	Inverse variance weighted	-0.045	0.040	0.956	0.885-1.034	0.261	0.885	1.034
Tumor necrosis factor alpha levels	Inverse variance weighted	-0.004	0.058	0.996	0.889-1.116	0.941	0.889	1.116
Stromal-cell-derived factor 1 alpha levels	Inverse variance weighted	0.070	0.089	1.072	0.901-1.277	0.433	0.901	1.277
Stem cell growth factor beta levels	Inverse variance weighted	0.008	0.038	1.008	0.936-1.086	0.835	0.936	1.086
Stem cell factor levels	Inverse variance weighted	0.109	0.069	1.115	0.975-1.276	0.112	0.975	1.276
Interleukin-16 levels	Inverse variance weighted	0.010	0.035	1.010	0.943-1.082	0.772	0.943	1.082
RANTES levels	Inverse variance weighted	0.010	0.061	1.010	0.897-1.138	0.863	0.897	1.138
Platelet-derived growth factor BB levels	Inverse variance weighted	-0.011	0.050	0.989	0.897-1.092	0.833	0.897	1.092
Macrophage inflammatory protein 1b levels	Inverse variance weighted	-0.032	0.031	0.969	0.911-1.030	0.307	0.911	1.030
Macrophage inflammatory protein 1a levels	Inverse variance weighted	-0.024	0.056	0.976	0.875-1.089	0.666	0.875	1.089
Monokine induced by gamma interferon levels	Inverse variance weighted	-0.022	0.042	0.978	0.902-1.061	0.594	0.902	1.061
Macrophage colony stimulating factor levels	Inverse variance weighted	-0.009	0.052	0.992	0.895-1.099	0.871	0.895	1.099
Monocyte chemoattractant protein-3 levels	Inverse variance weighted	0.071	0.060	1.074	0.955-1.207	0.236	0.955	1.207
Monocyte chemoattractant protein-1 levels	Inverse variance weighted	0.107	0.054	1.113	1.000-1.238	0.050	1.000	1.238
Interleukin-12p70 levels	Inverse variance weighted	0.024	0.041	1.025	0.945-1.111	0.555	0.945	1.111
Interferon gamma-induced protein 10 levels	Inverse variance weighted	0.026	0.055	1.026	0.922-1.143	0.636	0.922	1.143
Interleukin-18 levels	Inverse variance weighted	-0.007	0.038	0.993	0.922-1.070	0.857	0.922	1.070
Interleukin-17 levels	Inverse variance weighted	-0.085	0.070	0.919	0.802-1.053	0.224	0.802	1.053
Interleukin-13 levels	Inverse variance weighted	0.026	0.034	1.027	0.960-1.097	0.439	0.960	1.097
Interleukin-10 levels	Inverse variance weighted	0.004	0.061	1.004	0.891-1.131	0.947	0.891	1.131
Interleukin-8 levels	Inverse variance weighted	0.027	0.085	1.028	0.869-1.215	0.751	0.869	1.215
Interleukin-6 levels	Inverse variance weighted	0.018	0.098	1.018	0.840-1.233	0.857	0.840	1.233
Interleukin-1-receptor antagonist levels	Inverse variance weighted	0.023	0.061	1.023	0.907-1.154	0.713	0.907	1.154
Interleukin-1-beta levels	Inverse variance weighted	-0.017	0.084	0.983	0.834-1.160	0.842	0.834	1.160
Hepatocyte growth factor levels	Inverse variance weighted	-0.065	0.071	0.937	0.815-1.077	0.359	0.815	1.077
Interleukin-9 levels	Inverse variance weighted	-0.041	0.065	0.960	0.845-1.091	0.531	0.845	1.091
Interleukin-7 levels	Inverse variance weighted	0.038	0.038	1.039	0.964-1.119	0.319	0.964	1.119
Interleukin-5 levels	Inverse variance weighted	0.025	0.067	1.025	0.900-1.169	0.708	0.900	1.169
Interleukin-4 levels	Inverse variance weighted	0.040	0.080	1.041	0.890-1.218	0.616	0.890	1.218
Interleukin-2 receptor antagonist levels	Inverse variance weighted	0.037	0.039	1.038	0.961-1.120	0.345	0.961	1.120
Interleukin-2 levels	Inverse variance weighted	-0.014	0.056	0.986	0.883-1.101	0.805	0.883	1.101
Interferon gamma levels	Inverse variance weighted	-0.140	0.083	0.869	0.739-1.022	0.090	0.739	1.022
Growth-regulated protein alpha levels	Inverse variance weighted	0.026	0.039	1.027	0.952-1.108	0.498	0.952	1.108
Granulocyte-colony stimulating factor levels	Inverse variance weighted	-0.004	0.098	0.996	0.821-1.207	0.964	0.821	1.207
Fibroblast growth factor basic levels	Inverse variance weighted	0.126	0.120	1.134	0.896-1.436	0.294	0.896	1.436
Eotaxin levels	Inverse variance weighted	0.046	0.046	1.047	0.957-1.145	0.318	0.957	1.145

Among the other 35 examined cytokines, none demonstrated significant correlation with lung cancer risk or pathological types in IVW or secondary MR analyses (**Tables 1**–**4**). Heterogeneity testing showed no significant heterogeneity for any of the cytokines (**Tables 5**–**8**). Across all cytokines, MR Egger regression consistently revealed no evidence of pleiotropy (**Tables 5**–**8**). MR-PRESSO outlier testing validated the significant MR findings, except for monocyte chemoattractant protein-3 (MCP-3) in lung cancer (including different pathologic types) (**Supplementary Tables 6**-**9**), and macrophage migration inhibitory factor (MIF) and IL-5 in small-cell lung carcinoma, where limited SNPs were available (**Supplementary Table 9**). Furthermore, Steiger p-values <0.05 verified the detected causal direction was correct for all cytokines (**Table 9**–**12**).

**Table 2:** Primary results of MR analysis on lung cancer.

	Method	Beta	SE	OR	95% CI	*P* -value	LOW	UP
CTACK levels	Inverse variance weighted	-0.056	0.033	0.946	0.887-1.009	0.090	0.887	1.009
beta-nerve growth factor levels	Inverse variance weighted	0.007	0.039	1.007	0.933-1.086	0.857	0.933	1.086
Vascular endothelial growth factor levels	Inverse variance weighted	0.019	0.021	1.019	0.978-1.062	0.369	0.978	1.062
Macrophage Migration Inhibitory Factor levels	Inverse variance weighted	-0.027	0.041	0.974	0.899-1.055	0.514	0.899	1.055
TRAIL levels	Inverse variance weighted	0.038	0.023	1.039	0.992-1.088	0.101	0.992	1.088
Tumor necrosis factor beta levels	Inverse variance weighted	0.017	0.025	1.017	0.969-1.068	0.484	0.969	1.068
Tumor necrosis factor alpha levels	Inverse variance weighted	-0.022	0.036	0.978	0.911-1.050	0.538	0.911	1.050
Stromal-cell-derived factor 1 alpha levels	Inverse variance weighted	0.050	0.057	1.051	0.941-1.175	0.379	0.941	1.175
Stem cell growth factor beta levels	Inverse variance weighted	0.033	0.029	1.033	0.976-1.094	0.260	0.976	1.094
Stem cell factor levels	Inverse variance weighted	0.086	0.048	1.090	0.992-1.198	0.073	0.992	1.198
Interleukin-16 levels	Inverse variance weighted	0.023	0.022	1.023	0.980-1.068	0.299	0.980	1.068
RANTES levels	Inverse variance weighted	-0.033	0.033	0.967	0.907-1.032	0.317	0.907	1.032
Platelet-derived growth factor BB levels	Inverse variance weighted	-0.062	0.035	0.940	0.878-1.006	0.072	0.878	1.006
Macrophage inflammatory protein 1b levels	Inverse variance weighted	-0.006	0.020	0.994	0.956-1.034	0.774	0.956	1.034
Macrophage inflammatory protein 1a levels	Inverse variance weighted	-0.028	0.035	0.972	0.907-1.042	0.422	0.907	1.042
Monokine induced by gamma interferon levels	Inverse variance weighted	-0.006	0.027	0.994	0.943-1.048	0.815	0.943	1.048
Macrophage colony stimulating factor levels	Inverse variance weighted	0.012	0.028	1.012	0.959-1.068	0.665	0.959	1.068
Monocyte chemoattractant protein-3 levels	Inverse variance weighted	0.003	0.038	1.003	0.931-1.079	0.947	0.931	1.079
Monocyte chemoattractant protein-1 levels	Inverse variance weighted	0.048	0.031	1.049	0.988-1.115	0.118	0.988	1.115
Interleukin-12p70 levels	Inverse variance weighted	0.040	0.026	1.041	0.989-1.096	0.126	0.989	1.096
Interferon gamma-induced protein 10 levels	Inverse variance weighted	-0.024	0.036	0.977	0.909-1.049	0.517	0.909	1.049
Interleukin-18 levels*	Inverse variance weighted	-0.059	0.025	0.942	0.897-0.990	0.018	0.897	0.990
Interleukin-17 levels	Inverse variance weighted	-0.008	0.044	0.992	0.911-1.081	0.861	0.911	1.081
Interleukin-13 levels	Inverse variance weighted	0.032	0.022	1.032	0.989-1.077	0.149	0.989	1.077
Interleukin-10 levels	Inverse variance weighted	0.017	0.037	1.018	0.946-1.094	0.639	0.946	1.094
Interleukin-8 levels	Inverse variance weighted	0.004	0.059	1.004	0.894-1.128	0.947	0.894	1.128
Interleukin-6 levels	Inverse variance weighted	0.061	0.061	1.063	0.944-1.197	0.313	0.944	1.197
Interleukin-1-receptor antagonist levels	Inverse variance weighted	0.024	0.039	1.024	0.949-1.104	0.543	0.949	1.104
Interleukin-1-beta levels	Inverse variance weighted	0.095	0.054	1.100	0.990-1.222	0.075	0.990	1.222
Hepatocyte growth factor levels	Inverse variance weighted	0.009	0.045	1.009	0.924-1.101	0.850	0.924	1.101
Interleukin-9 levels	Inverse variance weighted	-0.011	0.041	0.989	0.913-1.072	0.789	0.913	1.072
Interleukin-7 levels	Inverse variance weighted	0.025	0.024	1.026	0.978-1.075	0.294	0.978	1.075
Interleukin-5 levels	Inverse variance weighted	0.046	0.042	1.047	0.964-1.138	0.275	0.964	1.138
Interleukin-4 levels	Inverse variance weighted	-0.002	0.055	0.998	0.897-1.112	0.976	0.897	1.112
Interleukin-2 receptor antagonist levels	Inverse variance weighted	-0.027	0.025	0.973	0.927-1.021	0.268	0.927	1.021
Interleukin-2 levels	Inverse variance weighted	-0.019	0.033	0.981	0.921-1.047	0.568	0.921	1.047
Interferon gamma levels	Inverse variance weighted	0.010	0.056	1.010	0.905-1.127	0.859	0.905	1.127
Growth-regulated protein alpha levels	Inverse variance weighted	0.034	0.020	1.035	0.994-1.077	0.091	0.994	1.077
Granulocyte-colony stimulating factor levels	Inverse variance weighted	0.035	0.048	1.036	0.943-1.137	0.465	0.943	1.137
Fibroblast growth factor basic levels	Inverse variance weighted	0.085	0.076	1.088	0.937-1.264	0.268	0.937	1.264
Eotaxin levels*	Inverse variance weighted	0.059	0.029	1.061	1.002-1.123	0.043	1.002	1.123

**Table 3:** Primary results of MR analysis on lung adenocarcinoma.

	Method	Beta	SE	OR	95% CI	*P* -value	LOW	UP
CTACK levels	Inverse variance weighted	-0.010	0.048	0.990	0.902-1.087	0.838	0.902	1.087
beta-nerve growth factor levels	Inverse variance weighted	-0.029	0.053	0.972	0.875-1.079	0.593	0.875	1.079
Vascular endothelial growth factor levels	Inverse variance weighted	0.029	0.029	1.029	0.972-1.090	0.319	0.972	1.090
Macrophage Migration Inhibitory Factor levels	Inverse variance weighted	-0.018	0.055	0.982	0.881-1.093	0.737	0.881	1.093
TRAIL levels	Inverse variance weighted	0.031	0.028	1.032	0.976-1.090	0.270	0.976	1.090
Tumor necrosis factor beta levels	Inverse variance weighted	0.018	0.034	1.018	0.953-1.087	0.592	0.953	1.087
Tumor necrosis factor alpha levels	Inverse variance weighted	0.026	0.050	1.027	0.932-1.132	0.595	0.932	1.132
Stromal-cell-derived factor 1 alpha levels	Inverse variance weighted	-0.002	0.089	0.998	0.838-1.188	0.979	0.838	1.188
Stem cell growth factor beta levels	Inverse variance weighted	-0.022	0.037	0.978	0.910-1.052	0.554	0.910	1.052
Stem cell factor levels*	Inverse variance weighted	0.140	0.061	1.150	1.021-1.296	0.021	1.021	1.296
Interleukin-16 levels	Inverse variance weighted	0.000	0.031	1.000	0.942-1.063	0.992	0.942	1.063
RANTES levels	Inverse variance weighted	-0.020	0.057	0.980	0.877-1.095	0.719	0.877	1.095
Platelet-derived growth factor BB levels	Inverse variance weighted	-0.006	0.049	0.994	0.903-1.095	0.902	0.903	1.095
Macrophage inflammatory protein 1b levels	Inverse variance weighted	0.044	0.029	1.045	0.988-1.106	0.120	0.988	1.106
Macrophage inflammatory protein 1a levels	Inverse variance weighted	-0.037	0.049	0.964	0.876-1.060	0.447	0.876	1.060
Monokine induced by gamma interferon levels	Inverse variance weighted	0.033	0.034	1.033	0.966-1.105	0.337	0.966	1.105
Macrophage colony stimulating factor levels	Inverse variance weighted	0.017	0.038	1.017	0.945-1.095	0.648	0.945	1.095
Monocyte chemoattractant protein-3 levels	Inverse variance weighted	-0.007	0.059	0.993	0.885-1.114	0.905	0.885	1.114
Monocyte chemoattractant protein-1 levels	Inverse variance weighted	0.014	0.042	1.014	0.933-1.102	0.744	0.933	1.102
Interleukin-12p70 levels	Inverse variance weighted	0.047	0.037	1.048	0.975-1.126	0.201	0.975	1.126
Interferon gamma-induced protein 10 levels	Inverse variance weighted	-0.008	0.045	0.992	0.908-1.083	0.859	0.908	1.083
Interleukin-18 levels	Inverse variance weighted	-0.054	0.031	0.947	0.891-1.007	0.083	0.891	1.007
Interleukin-17 levels	Inverse variance weighted	-0.031	0.065	0.970	0.853-1.102	0.635	0.853	1.102
Interleukin-13 levels	Inverse variance weighted	0.028	0.031	1.028	0.967-1.093	0.371	0.967	1.093
Interleukin-10 levels	Inverse variance weighted	0.052	0.044	1.053	0.966-1.147	0.239	0.966	1.147
Interleukin-8 levels	Inverse variance weighted	0.012	0.069	1.012	0.885-1.158	0.858	0.885	1.158
Interleukin-6 levels	Inverse variance weighted	0.020	0.100	1.020	0.839-1.241	0.839	0.839	1.241
Interleukin-1-receptor antagonist levels	Inverse variance weighted	0.072	0.053	1.075	0.969-1.193	0.173	0.969	1.193
Interleukin-1-beta levels*	Inverse variance weighted	0.142	0.071	1.152	1.003-1.325	0.046	1.003	1.325
Hepatocyte growth factor levels	Inverse variance weighted	0.050	0.067	1.052	0.922-1.200	0.454	0.922	1.200
Interleukin-9 levels	Inverse variance weighted	-0.014	0.057	0.987	0.882-1.103	0.811	0.882	1.103
Interleukin-7 levels	Inverse variance weighted	0.005	0.034	1.005	0.941-1.075	0.874	0.941	1.075
Interleukin-5 levels	Inverse variance weighted	0.079	0.059	1.082	0.964-1.214	0.179	0.964	1.214
Interleukin-4 levels	Inverse variance weighted	0.030	0.060	1.031	0.916-1.160	0.613	0.916	1.160
Interleukin-2 receptor antagonist levels	Inverse variance weighted	-0.036	0.039	0.965	0.895-1.041	0.358	0.895	1.041
Interleukin-2 levels	Inverse variance weighted	-0.018	0.043	0.982	0.903-1.068	0.669	0.903	1.068
Interferon gamma levels	Inverse variance weighted	0.086	0.077	1.090	0.938-1.267	0.261	0.938	1.267
Growth-regulated protein alpha levels	Inverse variance weighted	0.008	0.028	1.008	0.954-1.065	0.774	0.954	1.065
Granulocyte-colony stimulating factor levels	Inverse variance weighted	0.115	0.065	1.122	0.988-1.273	0.075	0.988	1.273
Fibroblast growth factor basic levels	Inverse variance weighted	-0.039	0.143	0.962	0.727-1.273	0.785	0.727	1.273
Eotaxin levels	Inverse variance weighted	0.055	0.046	1.056	0.966-1.155	0.232	0.966	1.155

**Table 4:** Primary results of MR analysis on small cell lung carcinoma.

	Method	Beta	SE	OR	95% CI	*P* -value	LOW	UP
CTACK levels	Inverse variance weighted	-0.017	0.080	0.984	0.841-1.150	0.836	0.841	1.150
beta-nerve growth factor levels	Inverse variance weighted	-0.096	0.122	0.908	0.715-1.154	0.431	0.715	1.154
Vascular endothelial growth factor levels*	Inverse variance weighted	0.110	0.052	1.117	1.008-1.237	0.035	1.008	1.237
Macrophage Migration Inhibitory Factor levels	Inverse variance weighted	-0.130	0.137	0.878	0.671-1.150	0.345	0.671	1.150
TRAIL levels	Inverse variance weighted	0.012	0.055	1.012	0.908-1.128	0.825	0.908	1.128
Tumor necrosis factor beta levels	Inverse variance weighted	0.096	0.062	1.100	0.975-1.242	0.122	0.975	1.242
Tumor necrosis factor alpha levels	Inverse variance weighted	0.031	0.109	1.031	0.833-1.276	0.777	0.833	1.276
Stromal-cell-derived factor 1 alpha levels	Inverse variance weighted	0.073	0.144	1.075	0.811-1.425	0.613	0.811	1.425
Stem cell growth factor beta levels	Inverse variance weighted	-0.127	0.071	0.881	0.767-1.012	0.074	0.767	1.012
Stem cell factor levels	Inverse variance weighted	0.038	0.117	1.039	0.826-1.306	0.746	0.826	1.306
Interleukin-16 levels	Inverse variance weighted	-0.064	0.064	0.938	0.827-1.064	0.321	0.827	1.064
RANTES levels*	Inverse variance weighted	-0.083	0.101	0.921	0.755-1.122	0.412	0.755	1.122
Platelet-derived growth factor BB levels	Inverse variance weighted	0.130	0.086	1.139	0.963-1.348	0.130	0.963	1.348
Macrophage inflammatory protein 1b levels	Inverse variance weighted	0.059	0.050	1.060	0.961-1.170	0.243	0.961	1.170
Macrophage inflammatory protein 1a levels	Inverse variance weighted	-0.074	0.092	0.929	0.776-1.111	0.418	0.776	1.111
Monokine induced by gamma interferon levels	Inverse variance weighted	-0.126	0.077	0.881	0.758-1.025	0.101	0.758	1.025
Macrophage colony stimulating factor levels	Inverse variance weighted	-0.065	0.107	0.937	0.760-1.156	0.545	0.760	1.156
Monocyte chemoattractant protein-3 levels	Inverse variance weighted	-0.049	0.105	0.952	0.775-1.169	0.640	0.775	1.169
Monocyte chemoattractant protein-1 levels	Inverse variance weighted	0.052	0.081	1.054	0.900-1.234	0.515	0.900	1.234
Interleukin-12p70 levels	Inverse variance weighted	-0.172	0.127	0.842	0.657-1.080	0.175	0.657	1.080
Interferon gamma-induced protein 10 levels	Inverse variance weighted	0.026	0.113	1.026	0.823-1.280	0.819	0.823	1.280
Interleukin-18 levels	Inverse variance weighted	0.020	0.095	1.021	0.847-1.230	0.829	0.847	1.230
Interleukin-17 levels	Inverse variance weighted	0.134	0.125	1.144	0.895-1.462	0.284	0.895	1.462
Interleukin-13 levels	Inverse variance weighted	0.045	0.061	1.046	0.929-1.178	0.457	0.929	1.178
Interleukin-10 levels	Inverse variance weighted	0.088	0.091	1.092	0.914-1.305	0.331	0.914	1.305
Interleukin-8 levels	Inverse variance weighted	-0.026	0.101	0.974	0.798-1.188	0.796	0.798	1.188
Interleukin-6 levels	Inverse variance weighted	-0.262	0.152	0.770	0.571-1.037	0.085	0.571	1.037
Interleukin-1-receptor antagonist levels	Inverse variance weighted	-0.003	0.095	0.997	0.828-1.200	0.974	0.828	1.200
Interleukin-1-beta levels	Inverse variance weighted	0.146	0.150	1.157	0.863-1.552	0.329	0.863	1.552
Hepatocyte growth factor levels	Inverse variance weighted	0.100	0.112	1.105	0.886-1.377	0.375	0.886	1.377
Interleukin-9 levels	Inverse variance weighted	-0.150	0.134	0.860	0.662-1.118	0.261	0.662	1.118
Interleukin-7 levels	Inverse variance weighted	0.098	0.081	1.102	0.941-1.292	0.227	0.941	1.292
Interleukin-5 levels	Inverse variance weighted	-0.292	0.171	0.747	0.534-1.044	0.088	0.534	1.044
Interleukin-4 levels	Inverse variance weighted	-0.157	0.165	0.854	0.619-1.179	0.339	0.619	1.179
Interleukin-2 receptor antagonist levels	Inverse variance weighted	0.025	0.062	1.026	0.908-1.159	0.685	0.908	1.159
Interleukin-2 levels	Inverse variance weighted	-0.031	0.081	0.970	0.828-1.136	0.702	0.828	1.136
Interferon gamma levels	Inverse variance weighted	0.063	0.129	1.065	0.827-1.372	0.627	0.827	1.372
Growth-regulated protein alpha levels	Inverse variance weighted	-0.021	0.050	0.979	0.887-1.080	0.672	0.887	1.080
Granulocyte-colony stimulating factor levels	Inverse variance weighted	-0.067	0.124	0.936	0.733-1.194	0.592	0.733	1.194
Fibroblast growth factor basic levels	Inverse variance weighted	0.227	0.221	1.255	0.814-1.936	0.305	0.814	1.936
Eotaxin levels	Inverse variance weighted	0.093	0.074	1.098	0.950-1.268	0.205	0.950	1.268

**Table 5:** Heterogeneity and pleiotropy analyses for lung cancer.

	Heterogenity		MR-Egger intercept		
	Q	Q_*P* -value	Egger_intercept	SE	*P* -value
CTACK levels	9.467	0.221	0.015	0.017	0.437
beta-nerve growth factor levels	1.972	0.922	-0.010	0.025	0.701
Vascular endothelial growth factor levels	4.633	0.865	-0.002	0.008	0.837
Macrophage Migration Inhibitory Factor levels	5.169	0.396	-0.010	0.022	0.680
TRAIL levels	18.476	0.186	0.017	0.008	0.058
Tumor necrosis factor beta levels	1.368	0.713	0.001	0.012	0.958
Tumor necrosis factor alpha levels	1.520	0.823	0.008	0.013	0.601
Stromal-cell-derived factor 1 alpha levels	3.251	0.861	0.000	0.012	0.969
Stem cell growth factor beta levels	4.410	0.818	-0.011	0.013	0.455
Stem cell factor levels	10.468	0.234	0.015	0.013	0.297
Interleukin-16 levels	3.888	0.867	-0.005	0.012	0.702
RANTES levels	5.089	0.748	-0.023	0.019	0.259
Platelet-derived growth factor BB levels	4.714	0.909	0.001	0.009	0.881
Macrophage inflammatory protein 1b levels	10.345	0.848	0.010	0.007	0.175
Macrophage inflammatory protein 1a levels	7.054	0.531	-0.016	0.017	0.387
Monokine induced by gamma interferon levels	6.754	0.819	0.001	0.013	0.940
Macrophage colony stimulating factor levels	3.157	0.870	-0.002	0.016	0.898
Monocyte chemoattractant protein-3 levels	0.650	0.722	0.024	0.038	0.643
Monocyte chemoattractant protein-1 levels	9.702	0.718	0.011	0.009	0.272
Interleukin-12p70 levels	8.763	0.459	-0.006	0.008	0.513
Interferon gamma-induced protein 10 levels	4.649	0.703	-0.007	0.014	0.611
Interleukin-18 levels	17.701	0.169	-0.009	0.011	0.436
Interleukin-17 levels	7.267	0.609	0.005	0.015	0.753
Interleukin-13 levels	8.197	0.414	-0.001	0.011	0.952
Interleukin-10 levels	12.221	0.201	-0.010	0.010	0.350
Interleukin-8 levels	6.748	0.080	-0.001	0.022	0.971
Interleukin-6 levels	1.884	0.597	0.026	0.030	0.481
Interleukin-1-receptor antagonist levels	3.493	0.745	-0.001	0.017	0.975
Interleukin-1-beta levels	1.915	0.751	0.011	0.017	0.555
Hepatocyte growth factor levels	5.229	0.515	0.011	0.016	0.534
Interleukin-9 levels	2.313	0.804	0.003	0.023	0.912
Interleukin-7 levels	9.032	0.434	0.034	0.018	0.088
Interleukin-5 levels	3.390	0.495	0.000	0.020	0.986
Interleukin-4 levels	10.638	0.155	0.008	0.017	0.662
Interleukin-2 receptor antagonist levels	3.991	0.678	0.002	0.013	0.868
Interleukin-2 levels	10.179	0.336	-0.013	0.011	0.244
Interferon gamma levels	12.365	0.193	0.004	0.016	0.821
Growth-regulated protein alpha levels	5.711	0.680	0.006	0.015	0.711
Granulocyte-colony stimulating factor levels	1.606	0.952	-0.004	0.010	0.740
Fibroblast growth factor basic levels	0.950	0.917	0.015	0.032	0.667
Eotaxin levels	13.944	0.530	0.005	0.010	0.592

**Table 6:** Heterogeneity and pleiotropy analyses for squamous cell lung cancer.

	Heterogenity		MR-Egger intercept		
	Q	Q_*P* -value	Egger_intercept	SE	*P* -value
CTACK levels	5.294	0.624	0.014	0.022	0.557
beta-nerve growth factor levels	11.842	0.106	0.009	0.052	0.876
Vascular endothelial growth factor levels	5.924	0.748	-0.015	0.013	0.286
Macrophage Migration Inhibitory Factor levels	2.979	0.703	0.018	0.031	0.605
TRAIL levels	8.324	0.759	0.014	0.014	0.358
Tumor necrosis factor beta levels	0.512	0.916	-0.002	0.019	0.908
Tumor necrosis factor alpha levels	3.773	0.438	-0.024	0.021	0.336
Stromal-cell-derived factor 1 alpha levels	5.994	0.540	0.018	0.019	0.395
Stem cell growth factor beta levels	11.684	0.554	-0.015	0.015	0.343
Stem cell factor levels	8.546	0.382	0.012	0.020	0.579
Interleukin-16 levels	6.337	0.609	0.005	0.018	0.801
RANTES levels	3.356	0.763	0.023	0.037	0.568
Platelet-derived growth factor BB levels	7.831	0.645	0.015	0.014	0.319
Macrophage inflammatory protein 1b levels	13.830	0.679	0.005	0.011	0.667
Macrophage inflammatory protein 1a levels	6.414	0.601	-0.012	0.027	0.670
Monokine induced by gamma interferon levels	8.590	0.737	-0.024	0.020	0.261
Macrophage colony stimulating factor levels	9.963	0.191	0.002	0.033	0.943
Monocyte chemoattractant protein-3 levels	1.810	0.405	-0.005	0.083	0.960
Monocyte chemoattractant protein-1 levels	9.848	0.544	0.019	0.017	0.302
Interleukin-12p70 levels	6.245	0.715	0.004	0.013	0.740
Interferon gamma-induced protein 10 levels	6.812	0.557	-0.017	0.019	0.412
Interleukin-18 levels	18.226	0.197	-0.018	0.016	0.286
Interleukin-17 levels	2.011	0.991	0.008	0.023	0.739
Interleukin-13 levels	7.260	0.509	0.008	0.016	0.625
Interleukin-10 levels	13.218	0.153	-0.013	0.017	0.480
Interleukin-8 levels	5.543	0.136	0.026	0.026	0.435
Interleukin-6 levels	2.706	0.439	0.054	0.048	0.382
Interleukin-1-receptor antagonist levels	1.267	0.973	0.002	0.027	0.950
Interleukin-1-beta levels	2.766	0.598	0.038	0.027	0.252
Hepatocyte growth factor levels	4.932	0.553	-0.010	0.026	0.723
Interleukin-9 levels	3.116	0.682	0.012	0.037	0.767
Interleukin-7 levels	6.327	0.707	0.051	0.028	0.105
Interleukin-5 levels	2.325	0.676	0.002	0.030	0.955
Interleukin-4 levels	6.443	0.375	-0.017	0.024	0.510
Interleukin-2 receptor antagonist levels	3.367	0.762	0.010	0.021	0.649
Interleukin-2 levels	9.627	0.292	-0.023	0.019	0.254
Interferon gamma levels	10.736	0.294	0.029	0.021	0.206
Growth-regulated protein alpha levels	14.379	0.109	0.043	0.027	0.150
Granulocyte-colony stimulating factor levels	11.928	0.103	-0.005	0.023	0.826
Fibroblast growth factor basic levels	1.814	0.770	-0.009	0.049	0.865
Eotaxin levels	11.077	0.747	0.013	0.016	0.437

**Table 7:** Heterogeneity and pleiotropy analyses for lung adenocarcinoma.

	Heterogenity		MR-Egger intercept		
	Q	Q_*P* -value	Egger_intercept	SE	*P* -value
CTACK levels	7.251	0.298	0.000	0.026	0.991
beta-nerve growth factor levels	2.263	0.894	-0.014	0.035	0.710
Vascular endothelial growth factor levels	7.809	0.553	0.004	0.012	0.737
Macrophage Migration Inhibitory Factor levels	1.214	0.944	-0.014	0.027	0.635
TRAIL levels	10.848	0.698	0.016	0.011	0.158
Tumor necrosis factor beta levels	0.939	0.816	-0.010	0.016	0.597
Tumor necrosis factor alpha levels	2.348	0.672	0.006	0.019	0.760
Stromal-cell-derived factor 1 alpha levels	9.372	0.227	-0.019	0.019	0.351
Stem cell growth factor beta levels	9.863	0.453	-0.022	0.015	0.193
Stem cell factor levels	8.867	0.354	0.014	0.017	0.444
Interleukin-16 levels	9.775	0.369	-0.009	0.015	0.560
RANTES levels	12.260	0.140	-0.067	0.026	0.037
Platelet-derived growth factor BB levels	16.557	0.167	-0.004	0.015	0.809
Macrophage inflammatory protein 1b levels	18.755	0.343	0.018	0.010	0.081
Macrophage inflammatory protein 1a levels	8.029	0.431	-0.038	0.023	0.147
Monokine induced by gamma interferon levels	8.394	0.817	-0.010	0.018	0.579
Macrophage colony stimulating factor levels	2.221	0.947	-0.024	0.023	0.323
Monocyte chemoattractant protein-3 levels	2.488	0.288	0.059	0.063	0.519
Monocyte chemoattractant protein-1 levels	12.510	0.486	0.001	0.013	0.969
Interleukin-12p70 levels	9.323	0.408	0.004	0.012	0.753
Interferon gamma-induced protein 10 levels	3.988	0.912	-0.003	0.016	0.865
Interleukin-18 levels	11.433	0.575	-0.002	0.012	0.866
Interleukin-17 levels	10.639	0.301	0.010	0.023	0.690
Interleukin-13 levels	1.102	0.954	-0.014	0.017	0.456
Interleukin-10 levels	7.209	0.615	0.001	0.012	0.915
Interleukin-8 levels	4.814	0.186	0.009	0.025	0.746
Interleukin-6 levels	6.764	0.149	-0.020	0.031	0.566
Interleukin-1-receptor antagonist levels	5.711	0.456	0.008	0.025	0.759
Interleukin-1-beta levels	1.917	0.751	-0.019	0.023	0.470
Hepatocyte growth factor levels	7.194	0.303	0.006	0.027	0.829
Interleukin-9 levels	3.762	0.584	-0.008	0.032	0.821
Interleukin-7 levels	9.504	0.392	0.034	0.024	0.199
Interleukin-5 levels	2.866	0.580	0.007	0.026	0.818
Interleukin-4 levels	6.922	0.437	0.016	0.018	0.412
Interleukin-2 receptor antagonist levels	7.841	0.250	0.007	0.022	0.782
Interleukin-2 levels	9.385	0.403	0.008	0.015	0.585
Interferon gamma levels	12.272	0.198	-0.006	0.022	0.795
Growth-regulated protein alpha levels	10.026	0.348	0.003	0.023	0.914
Granulocyte-colony stimulating factor levels	4.951	0.666	-0.012	0.014	0.451
Fibroblast growth factor basic levels	7.358	0.118	0.044	0.064	0.545
Eotaxin levels	19.714	0.183	0.000	0.016	0.991

**Table 8:** Heterogeneity and pleiotropy analyses for small cell lung carcinoma.

	Heterogenity		MR-Egger intercept		
	Q	Q_*P* -value	Egger_intercept	SE	*P* -value
CTACK levels	9.017	0.251	0.007	0.041	0.878
beta-nerve growth factor levels	9.257	0.160	-0.044	0.080	0.608
Vascular endothelial growth factor levels	7.042	0.532	0.014	0.021	0.515
Macrophage Migration Inhibitory Factor levels	0.369	0.831	0.045	0.112	0.759
TRAIL levels	11.474	0.404	-0.029	0.020	0.170
Tumor necrosis factor beta levels	2.820	0.420	0.047	0.030	0.252
Tumor necrosis factor alpha levels	1.058	0.787	0.032	0.041	0.521
Stromal-cell-derived factor 1 alpha levels	4.972	0.547	-0.045	0.030	0.202
Stem cell growth factor beta levels	4.180	0.939	-0.008	0.030	0.803
Stem cell factor levels	6.004	0.539	0.004	0.039	0.929
Interleukin-16 levels	11.230	0.189	-0.042	0.028	0.176
RANTES levels	9.807	0.200	-0.038	0.059	0.540
Platelet-derived growth factor BB levels	15.546	0.213	-0.033	0.024	0.203
Macrophage inflammatory protein 1b levels	10.777	0.768	-0.006	0.018	0.750
Macrophage inflammatory protein 1a levels	6.605	0.471	0.035	0.044	0.455
Monokine induced by gamma interferon levels	10.474	0.313	-0.025	0.037	0.510
Macrophage colony stimulating factor levels	6.862	0.143	0.040	0.061	0.555
Monocyte chemoattractant protein-3 levels	0.179	0.672	Not Applicable	Not Applicable	Not Applicable
Monocyte chemoattractant protein-1 levels	11.512	0.486	0.003	0.027	0.921
Interleukin-12p70 levels	3.995	0.677	-0.031	0.029	0.327
Interferon gamma-induced protein 10 levels	8.625	0.196	-0.018	0.049	0.734
Interleukin-18 levels	13.770	0.088	-0.078	0.037	0.076
Interleukin-17 levels	6.163	0.521	-0.014	0.048	0.780
Interleukin-13 levels	8.858	0.263	-0.017	0.030	0.593
Interleukin-10 levels	10.391	0.239	-0.041	0.023	0.114
Interleukin-8 levels	3.066	0.382	-0.035	0.031	0.378
Interleukin-6 levels	0.465	0.927	0.000	0.040	0.999
Interleukin-1-receptor antagonist levels	3.997	0.677	-0.012	0.042	0.778
Interleukin-1-beta levels	5.525	0.237	0.016	0.056	0.796
Hepatocyte growth factor levels	2.241	0.896	0.047	0.041	0.307
Interleukin-9 levels	5.782	0.216	-0.023	0.083	0.799
Interleukin-7 levels	11.503	0.118	-0.014	0.061	0.831
Interleukin-5 levels	0.932	0.334	Not Applicable	Not Applicable	Not Applicable
Interleukin-4 levels	2.089	0.719	-0.015	0.072	0.848
Interleukin-2 receptor antagonist levels	4.042	0.543	-0.020	0.032	0.563
Interleukin-2 levels	5.160	0.640	-0.012	0.026	0.663
Interferon gamma levels	2.416	0.878	-0.028	0.032	0.427
Growth-regulated protein alpha levels	3.733	0.880	-0.043	0.038	0.298
Granulocyte-colony stimulating factor levels	2.688	0.748	-0.030	0.026	0.318
Fibroblast growth factor basic levels	3.915	0.271	0.075	0.114	0.579
Eotaxin levels	5.365	0.966	-0.006	0.027	0.821

**Table 9:** Direction test for lung cancer.

Exposure	Outcome	Direction	Steiger *P* -value
CTACK levels	Lung cancer	TRUE	4.98E-84
beta-nerve growth factor levels	Lung cancer	TRUE	5.01E-46
Vascular endothelial growth factor levels	Lung cancer	TRUE	1.36E-259
Macrophage Migration Inhibitory Factor levels	Lung cancer	TRUE	8.47E-51
TRAIL levels	Lung cancer	TRUE	0
Tumor necrosis factor beta levels	Lung cancer	TRUE	5.71E-44
Tumor necrosis factor alpha levels	Lung cancer	TRUE	1.59E-25
Stromal-cell-derived factor 1 alpha levels	Lung cancer	TRUE	5.03E-34
Stem cell growth factor beta levels	Lung cancer	TRUE	2.72E-68
Stem cell factor levels	Lung cancer	TRUE	6.56E-58
Interleukin-16 levels	Lung cancer	TRUE	1.81E-83
RANTES levels	Lung cancer	TRUE	3.50E-60
Platelet-derived growth factor BB levels	Lung cancer	TRUE	2.61E-114
Macrophage inflammatory protein 1b levels	Lung cancer	TRUE	0
Macrophage inflammatory protein 1a levels	Lung cancer	TRUE	2.20E-41
Monokine induced by gamma interferon levels	Lung cancer	TRUE	8.51E-85
Macrophage colony stimulating factor levels	Lung cancer	TRUE	2.97E-60
Monocyte chemoattractant protein-3 levels	Lung cancer	TRUE	3.21E-28
Monocyte chemoattractant protein-1 levels	Lung cancer	TRUE	1.13E-123
Interleukin-12p70 levels	Lung cancer	TRUE	9.05E-301
Interferon gamma-induced protein 10 levels	Lung cancer	TRUE	5.14E-49
Interleukin-18 levels	Lung cancer	TRUE	2.64E-168
Interleukin-17 levels	Lung cancer	TRUE	1.32E-55
Interleukin-13 levels	Lung cancer	TRUE	2.99E-117
Interleukin-10 levels	Lung cancer	TRUE	1.09E-171
Interleukin-8 levels	Lung cancer	TRUE	6.92E-20
Interleukin-6 levels	Lung cancer	TRUE	1.71E-36
Interleukin-1-receptor antagonist levels	Lung cancer	TRUE	4.62E-43
Interleukin-1-beta levels	Lung cancer	TRUE	2.34E-23
Hepatocyte growth factor levels	Lung cancer	TRUE	6.94E-49
Interleukin-9 levels	Lung cancer	TRUE	1.66E-37
Interleukin-7 levels	Lung cancer	TRUE	6.40E-137
Interleukin-5 levels	Lung cancer	TRUE	8.87E-32
Interleukin-4 levels	Lung cancer	TRUE	7.50E-55
Interleukin-2 receptor antagonist levels	Lung cancer	TRUE	2.15E-73
Interleukin-2 levels	Lung cancer	TRUE	6.91E-75
Interferon gamma levels	Lung cancer	TRUE	2.44E-62
Growth-regulated protein alpha levels	Lung cancer	TRUE	2.94E-134
Granulocyte-colony stimulating factor levels	Lung cancer	TRUE	1.63E-56
Fibroblast growth factor basic levels	Lung cancer	TRUE	7.28E-36
Eotaxin levels	Lung cancer	TRUE	7.78E-136

**Table 10:** Direction test for squamous cell lung cancer.

Exposure	Outcome	Direction	Steiger *P* -value
CTACK levels	Squamous cell lung cancer	TRUE	7.19E-88
beta-nerve growth factor levels	Squamous cell lung cancer	TRUE	5.27E-50
Vascular endothelial growth factor levels	Squamous cell lung cancer	TRUE	1.35E-249
Macrophage Migration Inhibitory Factor levels	Squamous cell lung cancer	TRUE	3.75E-51
TRAIL levels	Squamous cell lung cancer	TRUE	2.88E-305
Tumor necrosis factor beta levels	Squamous cell lung cancer	TRUE	1.40E-43
Tumor necrosis factor alpha levels	Squamous cell lung cancer	TRUE	2.16E-24
Stromal-cell-derived factor 1 alpha levels	Squamous cell lung cancer	TRUE	7.87E-32
Stem cell growth factor beta levels	Squamous cell lung cancer	TRUE	3.92E-117
Stem cell factor levels	Squamous cell lung cancer	TRUE	3.81E-56
Interleukin-16 levels	Squamous cell lung cancer	TRUE	1.30E-81
RANTES levels	Squamous cell lung cancer	TRUE	4.19E-49
Platelet-derived growth factor BB levels	Squamous cell lung cancer	TRUE	8.27E-126
Macrophage inflammatory protein 1b levels	Squamous cell lung cancer	TRUE	0
Macrophage inflammatory protein 1a levels	Squamous cell lung cancer	TRUE	1.52E-40
Monokine induced by gamma interferon levels	Squamous cell lung cancer	TRUE	1.61E-86
Macrophage colony stimulating factor levels	Squamous cell lung cancer	TRUE	6.56E-59
Monocyte chemoattractant protein-3 levels	Squamous cell lung cancer	TRUE	1.84E-27
Monocyte chemoattractant protein-1 levels	Squamous cell lung cancer	TRUE	5.41E-92
Interleukin-12p70 levels	Squamous cell lung cancer	TRUE	3.46E-292
Interferon gamma-induced protein 10 levels	Squamous cell lung cancer	TRUE	1.23E-56
Interleukin-18 levels	Squamous cell lung cancer	TRUE	8.72E-175
Interleukin-17 levels	Squamous cell lung cancer	TRUE	1.23E-54
Interleukin-13 levels	Squamous cell lung cancer	TRUE	6.12E-117
Interleukin-10 levels	Squamous cell lung cancer	TRUE	3.66E-164
Interleukin-8 levels	Squamous cell lung cancer	TRUE	1.82E-19
Interleukin-6 levels	Squamous cell lung cancer	TRUE	3.56E-34
Interleukin-1-receptor antagonist levels	Squamous cell lung cancer	TRUE	1.81E-42
Interleukin-1-beta levels	Squamous cell lung cancer	TRUE	5.46E-23
Hepatocyte growth factor levels	Squamous cell lung cancer	TRUE	6.73E-46
Interleukin-9 levels	Squamous cell lung cancer	TRUE	8.94E-36
Interleukin-7 levels	Squamous cell lung cancer	TRUE	1.19E-134
Interleukin-5 levels	Squamous cell lung cancer	TRUE	6.16E-32
Interleukin-4 levels	Squamous cell lung cancer	TRUE	9.47E-48
Interleukin-2 receptor antagonist levels	Squamous cell lung cancer	TRUE	1.16E-70
Interleukin-2 levels	Squamous cell lung cancer	TRUE	1.03E-63
Interferon gamma levels	Squamous cell lung cancer	TRUE	2.43E-60
Growth-regulated protein alpha levels	Squamous cell lung cancer	TRUE	2.47E-134
Granulocyte-colony stimulating factor levels	Squamous cell lung cancer	TRUE	1.86E-53
Fibroblast growth factor basic levels	Squamous cell lung cancer	TRUE	2.52E-34
Eotaxin levels	Squamous cell lung cancer	TRUE	2.44E-132

**Table 11:** Direction test for lung adenocarcinoma.

Exposure	Outcome	Direction	Steiger *P* -value
CTACK levels	Lung adenocarcinoma	TRUE	5.14E-77
beta-nerve growth factor levels	Lung adenocarcinoma	TRUE	4.85E-45
Vascular endothelial growth factor levels	Lung adenocarcinoma	TRUE	3.62E-252
Macrophage Migration Inhibitory Factor levels	Lung adenocarcinoma	TRUE	5.14E-51
TRAIL levels	Lung adenocarcinoma	TRUE	0
Tumor necrosis factor beta levels	Lung adenocarcinoma	TRUE	8.71E-44
Tumor necrosis factor alpha levels	Lung adenocarcinoma	TRUE	7.78E-25
Stromal-cell-derived factor 1 alpha levels	Lung adenocarcinoma	TRUE	2.21E-31
Stem cell growth factor beta levels	Lung adenocarcinoma	TRUE	2.98E-80
Stem cell factor levels	Lung adenocarcinoma	TRUE	8.86E-56
Interleukin-16 levels	Lung adenocarcinoma	TRUE	1.02E-88
RANTES levels	Lung adenocarcinoma	TRUE	1.95E-57
Platelet-derived growth factor BB levels	Lung adenocarcinoma	TRUE	1.29E-131
Macrophage inflammatory protein 1b levels	Lung adenocarcinoma	TRUE	0
Macrophage inflammatory protein 1a levels	Lung adenocarcinoma	TRUE	2.90E-40
Monokine induced by gamma interferon levels	Lung adenocarcinoma	TRUE	6.90E-95
Macrophage colony stimulating factor levels	Lung adenocarcinoma	TRUE	6.96E-60
Monocyte chemoattractant protein-3 levels	Lung adenocarcinoma	TRUE	1.38E-27
Monocyte chemoattractant protein-1 levels	Lung adenocarcinoma	TRUE	1.09E-118
Interleukin-12p70 levels	Lung adenocarcinoma	TRUE	4.36E-292
Interferon gamma-induced protein 10 levels	Lung adenocarcinoma	TRUE	7.43E-64
Interleukin-18 levels	Lung adenocarcinoma	TRUE	7.81E-148
Interleukin-17 levels	Lung adenocarcinoma	TRUE	5.41E-52
Interleukin-13 levels	Lung adenocarcinoma	TRUE	2.06E-100
Interleukin-10 levels	Lung adenocarcinoma	TRUE	1.53E-168
Interleukin-8 levels	Lung adenocarcinoma	TRUE	9.66E-20
Interleukin-6 levels	Lung adenocarcinoma	TRUE	6.65E-41
Interleukin-1-receptor antagonist levels	Lung adenocarcinoma	TRUE	3.88E-41
Interleukin-1-beta levels	Lung adenocarcinoma	TRUE	7.61E-23
Hepatocyte growth factor levels	Lung adenocarcinoma	TRUE	1.80E-45
Interleukin-9 levels	Lung adenocarcinoma	TRUE	1.12E-36
Interleukin-7 levels	Lung adenocarcinoma	TRUE	1.17E-134
Interleukin-5 levels	Lung adenocarcinoma	TRUE	1.99E-31
Interleukin-4 levels	Lung adenocarcinoma	TRUE	1.87E-54
Interleukin-2 receptor antagonist levels	Lung adenocarcinoma	TRUE	1.61E-70
Interleukin-2 levels	Lung adenocarcinoma	TRUE	6.07E-68
Interferon gamma levels	Lung adenocarcinoma	TRUE	1.79E-60
Growth-regulated protein alpha levels	Lung adenocarcinoma	TRUE	8.76E-137
Granulocyte-colony stimulating factor levels	Lung adenocarcinoma	TRUE	2.31E-54
Fibroblast growth factor basic levels	Lung adenocarcinoma	TRUE	5.71E-32
Eotaxin levels	Lung adenocarcinoma	TRUE	7.40E-130

**Table 12:** Direction test for small cell lung carcinoma.

Exposure	Outcome	Direction	Steiger *P* -value
CTACK levels	Small cell lung carcinoma	TRUE	7.50E-69
beta-nerve growth factor levels	Small cell lung carcinoma	TRUE	3.14E-36
Vascular endothelial growth factor levels	Small cell lung carcinoma	TRUE	1.02E-192
Macrophage Migration Inhibitory Factor levels	Small cell lung carcinoma	TRUE	2.27E-32
TRAIL levels	Small cell lung carcinoma	TRUE	4.2579E-238
Tumor necrosis factor beta levels	Small cell lung carcinoma	TRUE	1.74E-34
Tumor necrosis factor alpha levels	Small cell lung carcinoma	TRUE	4.40E-18
Stromal-cell-derived factor 1 alpha levels	Small cell lung carcinoma	TRUE	1.97E-24
Stem cell growth factor beta levels	Small cell lung carcinoma	TRUE	4.40E-66
Stem cell factor levels	Small cell lung carcinoma	TRUE	1.77E-40
Interleukin-16 levels	Small cell lung carcinoma	TRUE	3.26E-66
RANTES levels	Small cell lung carcinoma	TRUE	2.70E-45
Platelet-derived growth factor BB levels	Small cell lung carcinoma	TRUE	5.20E-94
Macrophage inflammatory protein 1b levels	Small cell lung carcinoma	TRUE	0
Macrophage inflammatory protein 1a levels	Small cell lung carcinoma	TRUE	3.25E-31
Monokine induced by gamma interferon levels	Small cell lung carcinoma	TRUE	1.34E-58
Macrophage colony stimulating factor levels	Small cell lung carcinoma	TRUE	3.30E-36
Monocyte chemoattractant protein-3 levels	Small cell lung carcinoma	TRUE	1.28E-21
Monocyte chemoattractant protein-1 levels	Small cell lung carcinoma	TRUE	5.72E-87
Interleukin-12p70 levels	Small cell lung carcinoma	TRUE	7.14E-124
Interferon gamma-induced protein 10 levels	Small cell lung carcinoma	TRUE	6.96E-41
Interleukin-18 levels	Small cell lung carcinoma	TRUE	2.86E-72
Interleukin-17 levels	Small cell lung carcinoma	TRUE	1.74E-33
Interleukin-13 levels	Small cell lung carcinoma	TRUE	1.07E-86
Interleukin-10 levels	Small cell lung carcinoma	TRUE	6.85E-126
Interleukin-8 levels	Small cell lung carcinoma	TRUE	1.40E-17
Interleukin-6 levels	Small cell lung carcinoma	TRUE	1.19E-29
Interleukin-1-receptor antagonist levels	Small cell lung carcinoma	TRUE	2.03E-28
Interleukin-1-beta levels	Small cell lung carcinoma	TRUE	1.65E-19
Hepatocyte growth factor levels	Small cell lung carcinoma	TRUE	4.00E-35
Interleukin-9 levels	Small cell lung carcinoma	TRUE	4.27E-27
Interleukin-7 levels	Small cell lung carcinoma	TRUE	2.99E-99
Interleukin-5 levels	Small cell lung carcinoma	TRUE	6.73E-12
Interleukin-4 levels	Small cell lung carcinoma	TRUE	1.19E-29
Interleukin-2 receptor antagonist levels	Small cell lung carcinoma	TRUE	1.61E-59
Interleukin-2 levels	Small cell lung carcinoma	TRUE	1.52E-54
Interferon gamma levels	Small cell lung carcinoma	TRUE	1.09E-39
Growth-regulated protein alpha levels	Small cell lung carcinoma	TRUE	3.86E-120
Granulocyte-colony stimulating factor levels	Small cell lung carcinoma	TRUE	1.10E-24
Fibroblast growth factor basic levels	Small cell lung carcinoma	TRUE	1.75E-18
Eotaxin levels	Small cell lung carcinoma	TRUE	2.07E-98

### Subgroup analyses

Additional subgroup analyses stratified by smoking status were conducted to investigate whether the causal effect of cytokines on lung cancer risk was modified by smoking. In ever smokers, we found that interferon gamma-induced protein 10 (IP-10) (OR = 0.861, 95% CI: 0.781-0.950, P = 0.003) was inversely associated with lung cancer risk, while IL-1β (OR = 1.190, 95% CI: 1.023-1.384, P = 0.024) was positively associated with lung cancer risk (**Supplementary Tables 10**, **11**). Scatter plots visualized the effects for each method across datasets (**Supplementary Figures 7**, **8**). Heterogeneity tests showed no significant heterogeneity across cytokines, except for tumor necrosis factor (TNF)-α (**Supplementary Table 12**). MR-Egger regression revealed no evidence of horizontal pleiotropy for any cytokine (**Supplementary Table 12**). The MR-PRESSO outlier test confirmed significant MR findings, except for TNF-β, TNF-α, and MCP-3, due to limited SNP availability (**Supplementary Table 13**). Leave-one-out analysis confirmed the robustness of the causal estimates (**Supplementary Figures 9**, **10**). Furthermore, Steiger p-values <0.05 verified the correct causal direction for all detected cytokines (**Supplementary Table 14**).

Among never smokers, positive association was observed between lung cancer risk and SCF (OR = 1.474, 95% CI: 1.105-1.964, P = 0.008) (**Supplementary Tables 15**, **16**). Scatter plots visualized the effects for each method across datasets (**Supplementary Figure 11**). Heterogeneity was non-significant across all cytokines (**Supplementary Table 17**). MR-Egger and MR-PRESSO global tests showed no evidence of pleiotropy (**Supplementary Tables 17**, **18**). The MR-PRESSO outlier test confirmed significant MR findings, except for MCP-3, IL-8, and fibroblast growth factor basic (FGF-basic), due to limited SNPs (**Supplementary Table 18**). Leave-one-out analysis confirmed the robustness of the causal estimates (**Supplementary Figures 12**). Additionally, Steiger p-values <0.05 verified the expected causal direction for all detected cytokines (**Supplementary Table 19**).

## Discussion

In this MR study, we investigated potential causal relationships between 41 circulating cytokines and lung cancer risk. Our analysis provides preliminary evidence that genetically predicted levels of certain cytokines may influence cancer susceptibility. We identified four inflammatory mediators - SCF, IL-1β, IL-18, and IP-10 - involved in genetic susceptibility to lung cancer overall and in specific histologic subtypes, as well as differences based on smoking status. These results illuminate cytokine pathways that may promote cancer development, representing potential targets for prevention, early detection, and treatment. Our findings reveal putative causal effects of circulating cytokines in lung cancer pathogenesis and shed light on cytokine-mediated mechanisms influencing susceptibility across lung cancer subtypes and smoking strata.

Eotaxin, a chemokine, plays a pivotal role in managing a spectrum of inflammatory and immune-responsive conditions, chiefly by recruiting and activating eosinophils (30, 31). However, its specific function in lung cancer remains to be elucidated. Multiple studies have shown that eotaxin expression levels correlate with occurrence and prognosis in several cancers. Specifically, Yamaguchi et al. found lower eotaxin levels in healthy controls versus colon cancer patients, while Melisi et al. showed decreased eotaxin-2 levels in pancreatic cancer patients after treatment with a TGF-β receptor inhibitor and gemcitabine (32, 33). Additionally, Siva et al. reported reduced serum eotaxin-1 in non-small cell lung cancer patients following radiotherapy compared to radiotherapy alone, and Tsao et al. revealed an association between low serum eotaxin-1 and shorter progression-free survival in non-small cell lung cancer patients on vandetanib (34, 35). The statistical power of eotaxin was calculated to be only 67% and had a non-significant p-value after Bonferroni correction. In summary, although eotaxin is closely linked to pathogenesis and prognosis in some cancers, further research is warranted to elucidate its precise role in lung cancer. SCF activates the c-Kit signaling pathway to stimulate lung cancer cell proliferation, migration and invasion (36). *In vitro* experiments show SCF promotes lung cancer cell proliferation, migration and metastasis (37–39). Moreover, clinical studies find elevated SCF and c-Kit expression correlates with lung cancer progression and metastasis (40, 41). IL-1β advances lung cancer progression through numerous mechanisms including inducing angiogenic factors like VEGF, activating oncogenic signaling, and promoting immunosuppression and inflammation (42–44). Mechanistically, IL-1β spurs tumor growth by activating MAPK/NF-κB pathways, recruiting immunosuppressive cells, promoting inflammation, angiogenesis, invasion and metastasis, downregulating tumor suppressors, upregulating oncogenes, and enabling immune evasion and inhibition of apoptosis (45). Preclinical studies also demonstrate beneficial effects of IL-1β knockdown and inhibition. No inflammation or angiogenesis occurred in IL-1β-deficient mice, and IL-1β-deficient mice showed no local tumor growth or lung metastasis compared to wild-type mice (46). Moreover, IL-1β antibody treatment inhibited tumor progression and boosted antitumor immunity in mice by reducing inflammation and promoting M1 macrophage maturation (47). VEGF is a pivotal driver of angiogenesis in lung cancer, promoting tumor growth and metastasis (48). Furthermore, IL-1β induces VEGF secretion, which also spurs tumor expansion and spread (49). In lung cancer, VEGF overexpression critically supports angiogenesis and correlates with disease progression and prognosis (50). Multiple studies demonstrate VEGF inhibition slows lung cancer growth and improves chemotherapy efficacy (51, 52). Anti-VEGF monoclonal antibody therapies including bevacizumab are now widely used in lung cancer treatment (53). Similarly, the calculated statistical power of VEGF is only 21%.

In contrast, increased genetic predisposition to higher interleukin-18 (IL-18) and interferon gamma-induced protein 10 (IP-10) levels are negatively associated with lung cancer risk. IL-18, a pro-inflammatory cytokine, plays a significant role in anti-tumor immunity through several mechanisms. Firstly, IL-18 activates natural killer (NK) cells and certain subsets of T cells, enhancing their cytotoxic activity against tumor cells by upregulating cytotoxic molecules such as perforin and granzymes, which are crucial for direct tumor cell killing (54). Secondly, IL-18 stimulates the production of key anti-tumor cytokines, notably interferon-gamma (IFN-γ), which has potent anti-tumor effects, including the inhibition of tumor cell proliferation, induction of tumor cell apoptosis, and enhancement of antigen presentation to help the immune system better recognize and target tumor cells (55). Thirdly, IL-18 facilitates the infiltration of immune cells, particularly T cells and NK cells, into the tumor microenvironment, thereby enhancing the overall immune response against the tumor (56). Additionally, IL-18 promotes the differentiation of T cells towards a Th1 phenotype; Th1 cells produce IFN-γ and activate macrophages and other immune cells that contribute to anti-tumor immunity, making this shift towards a Th1-dominated response crucial for effective anti-tumor activity (57, 58). Furthermore, IL-18 often works synergistically with other cytokines and immune signals to amplify the immune response, significantly boosting IFN-γ production and enhancing the cytotoxic activity of NK cells and T cells when combined with IL-12 (59, 60). IL-18 also exhibits anti-angiogenic and pro-lymphangiogenic properties that contribute to its anti-tumor activity (57). However, Jiang et al. found that IL-18 may promote metastasis by inhibiting E-cadherin expression (58). Conversely, experimental studies by Xiong et al. and Chen et al. demonstrated that IL-18 inhibited tumor proliferation and growth, enhanced apoptosis, and normalized the Th1/Th2 imbalance (59, 60).

In contrast, increased genetic predisposition to higher levels of interleukin-18 (IL-18) and interferon gamma-induced protein 10 (IP-10) is negatively associated with lung cancer risk. IL-18, a pro-inflammatory cytokine, plays a significant role in anti-tumor immunity through several mechanisms. Firstly, IL-18 activates natural killer (NK) cells and certain subsets of T cells, enhancing their cytotoxic activity against tumor cells by upregulating cytotoxic molecules such as perforin and granzymes, which are crucial for direct tumor cell killing (54). Secondly, IL-18 stimulates the production of key anti-tumor cytokines, notably interferon-gamma (IFN-γ), which has potent anti-tumor effects, including the inhibition of tumor cell proliferation, induction of tumor cell apoptosis, and enhancement of antigen presentation to help the immune system better recognize and target tumor cells (55). Thirdly, IL-18 facilitates the infiltration of immune cells, particularly T cells and NK cells, into the tumor microenvironment, thereby enhancing the overall immune response against the tumor (56). Additionally, IL-18 promotes the differentiation of T cells towards a Th1 phenotype; Th1 cells produce IFN-γ and activate macrophages and other immune cells that contribute to anti-tumor immunity, making this shift towards a Th1-dominated response crucial for effective anti-tumor activity (57, 58). Finally, IL-18 often works synergistically with other cytokines and immune signals to amplify the immune response, significantly boosting IFN-γ production and enhancing the cytotoxic activity of NK cells and T cells when combined with IL-12 (59, 60). IL-18 also has anti-angiogenic and pro-lymphangiogenic properties contributing to its anti-tumor activity (61). However, Jiang et al. found that IL-18 may promote metastasis by inhibiting E-cadherin expression (62). Conversely, experimental studies by Xiong et al. and Chen et al. demonstrated that IL-18 inhibited tumor proliferation and growth, enhanced apoptosis, and normalized the Th1/Th2 imbalance (63, 64). IP-10 attracts immune cells to tumors, inhibits angiogenesis, and reduces tumor burden (65). Previous studies confirm IP-10 participates in anti-tumor immunity by promoting immune cell migration to tumors, inducing apoptosis, and suppressing angiogenesis (66–69). In xenograft models of lymphoma, squamous cell carcinoma, and lung adenocarcinoma, CXCL10 production negatively correlated with tumor growth and significantly reduced tumor-associated angiogenesis (70). In advanced endometrial cancer, CXCL10 was shown to antagonize fibroblast growth factor action, thereby inhibiting angiogenesis. In estrogen receptor-positive breast tumors, CXCL10 inhibits vascular endothelial growth factor levels to reduce tumor burden (71).

We utilized Mendelian randomization to evaluate associations between circulating cytokines and lung cancer risk. Contrary to a prior study by Bouras et al. showing positive CTACK-nonsmoking lung cancer and negative IL-18-lung cancer/adenocarcinoma relationships (72), we found no evidence for CTACK-nonsmoking lung cancer or IL-18-adenocarcinoma associations, potentially attributable to divergent instrument variable selection and GWAS data pooling. Notably, our analysis revealed novel causal links between SCF, IL-1β, IL-18, and IP-10 in overall lung cancer as well as specific histological subtypes, highlighting important etiological roles for these cytokines. However, some limitations should be considered. First, the relaxed IV significance threshold of P < 5×10^-6^ introduces possible false positives and bias, although the consistent F-statistics >10 suggest weak instrument bias is less likely. Second, the single Finnish ethnicity limits generalizability to other populations. Third, no cytokines were statistically significantly associated with cancer risk or subtypes after Bonferroni correction, including six inflammatory factors with suggestive correlations - Eotaxin, SCF, IL-1β, VEGF, IL-18, and IP-10. However, excluding Eotaxin and VEGF, the other factors (SCF, IL-1β, IL-18, and IP-10) had statistical power over 80% but still require validation of these potential associations in larger cohorts and GWAS. Fourth, while efforts were made to mitigate confounding, pleiotropy cannot be completely ruled out. Finally, while not addressed here, inflammatory factors may influence lung cancer progression and survival rather than development. Therefore, further studies should analyze the role of inflammatory factors in lung cancer aggressiveness.

## Conclusion

This MR study found preliminary evidence that genetically predicted levels of four inflammatory cytokines—SCF, IL-1β, IL-18, and IP-10—may causally influence lung cancer risk overall, in specific histologic subtypes, and stratified by smoking status. The identification of these cytokine pathways, which may promote lung carcinogenesis, represents potential new targets for the prevention, early detection, and treatment of lung cancer. Overall, these findings reveal putative causal effects of circulating cytokines in lung cancer pathogenesis, illuminating cytokine-mediated immunological mechanisms affecting susceptibility across lung cancer subtypes and smoking exposure groups.

## Data availability statement

The original contributions presented in the study are included in the article/**Supplementary Material**, further inquiries can be directed to the corresponding authors.

## Author contributions

DL: Data curation, Formal analysis, Writing – original draft. ZG: Data curation, Formal analysis, Writing – original draft. QZ: Data curation, Formal analysis, Writing – original draft, Writing – review & editing. SL: Conceptualization, Data curation, Formal analysis, Funding acquisition, Writing – original draft, Writing – review & editing.

## Supplementary material

Supplementary Figure 1 Scatter plot of IL-18 levels on lung cancer.

Supplementary Figure 2 Scatter plot of SCF levels on lung lung adenocarcinoma.

Supplementary Figure 3 Scatter plot of IL-1β levels on lung lung adenocarcinoma.

Supplementary Figure 4 Leave-one-out plot of IL-1β levels on lung cancer.

Supplementary Figure 5 Leave-one-out plot of SCF levels on lung adenocarcinoma.

Supplementary Figure 6 Leave-one-out plot of IL-1β levels on lung adenocarcinoma.

Supplementary Figure 7 Scatter plot of IP-10 levels on lung cancer in ever smokers.

Supplementary Figure 8 Scatter plot of IL-1β levels on lung cancer in ever smokers.

Supplementary Figure 9 Leave-one-out plot of IP-10 levels on lung cancer in ever smokers.

Supplementary Figure 10 Leave-one-out plot of IL-1β levels on lung cancer in ever smokers.

Supplementary Figure 11 Scatter plot of SCF levels on lung cancer in never smokers.

Supplementary Figure 12 Leave-one-out plot of SCF levels on lung cancer in never smokers.

## Abbreviations

**Table:**

GWAS	genome-wide association studies
SNPs	single nucleotide polymorphisms
IVs	instrumental variables
OR	odds ratio
CI	confidence interval
IVW	inverse variance weighting
MR-PRESSO	MR pleiotropy residual sum and outlier
ICD	International Classification of Diseases
MR	mendelian randomization
beta-NGF	beta nerve growth factor
CTACK	cutaneous T-cell attracting (CCL27)
FGF-basic	fibroblast growth factor basic
G-CSF	granulocyte colony-stimulating factor
GRO-alpha	growth regulated oncogene-alpha
HGF	hepatocyte growth factor
IFN-gamma	interferon-gamma
IL-1ra	interleukin-1 receptor antagonist
IL-1 beta	interleukin-1 beta
IL-2	interleukin-2
IL-2ra	interleukin-2 receptor antagonist
IL-4	interleukin-4
IL-5	interleukin-5
IL-6	interleukin-6
IL-7	interleukin-7
IL-8	interleukin-8
IL-9	interleukin-9
IL-10	interleukin-10
IL-12p70	interleukin-12p70
IL-13	interleukin-13
IL-16	interleukin-16
IL-17	interleukin-17
IL-18	interleukin-18
IP-10	interferon gamma-induced protein 10
MCP-1	monocyte chemoattractant protein-1
MCP-3	monocyte chemoattractant protein-3
M-CSF	macrophage colony-stimulating factor
MIF	macrophage migration inhibitory factor
MIG	monokine induced by gamma interferon
MIP-1a	macrophage inflammatory protein 1a
MIP-1b	macrophage inflammatory protein 1b
PDGF-bb	platelet-derived growth factor BB
RANTES	regulated on activation, normal T-cell expressed and secreted (CCL5)
SCF	stem cell factor
SCGF-beta	stem cell growth factor beta
SDF-1 alpha	stromal-cell-derived factor 1 alpha
TNF-alpha	tumor necrosis factor-alpha
TNF-beta	tumor necrosis factor-beta
TRAIL	TNF-related apoptosis inducing ligand
VEGF	vascular endothelial growth factor
