# Supplementary figures and images for: Causal association of circulating cytokines with the risk of lung cancer: a Mendelian randomization study
Source: Front Oncol. 2024 Jun 18;14:1373380. doi: 10.3389/fonc.2024.1373380 (PMC11217496; doi:10.3389/fonc.2024.1373380)

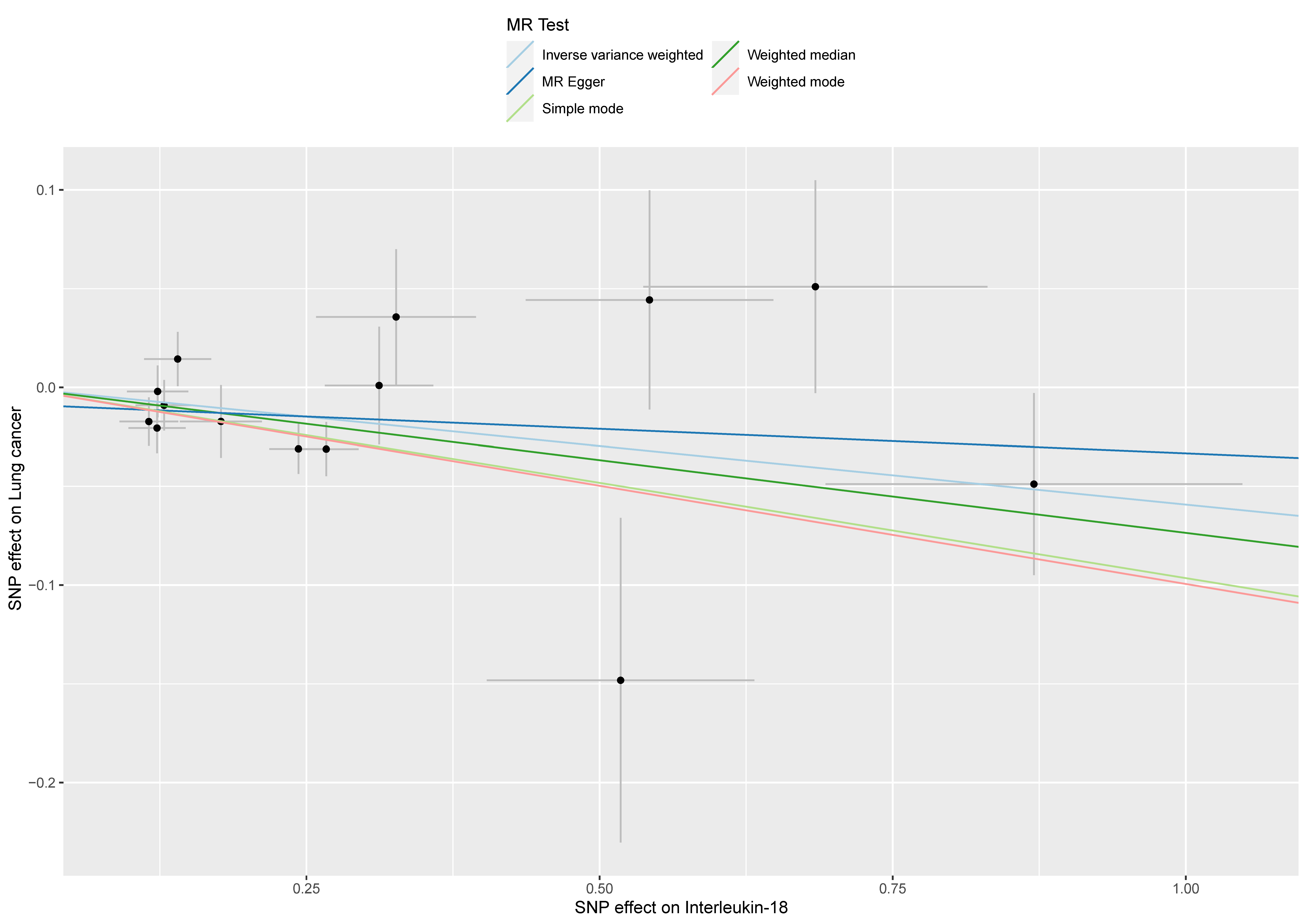

Supplement: Supplementary Figure 1 — Scatter plot of IL-18 levels on lung cancer. [file Image_1.tiff]

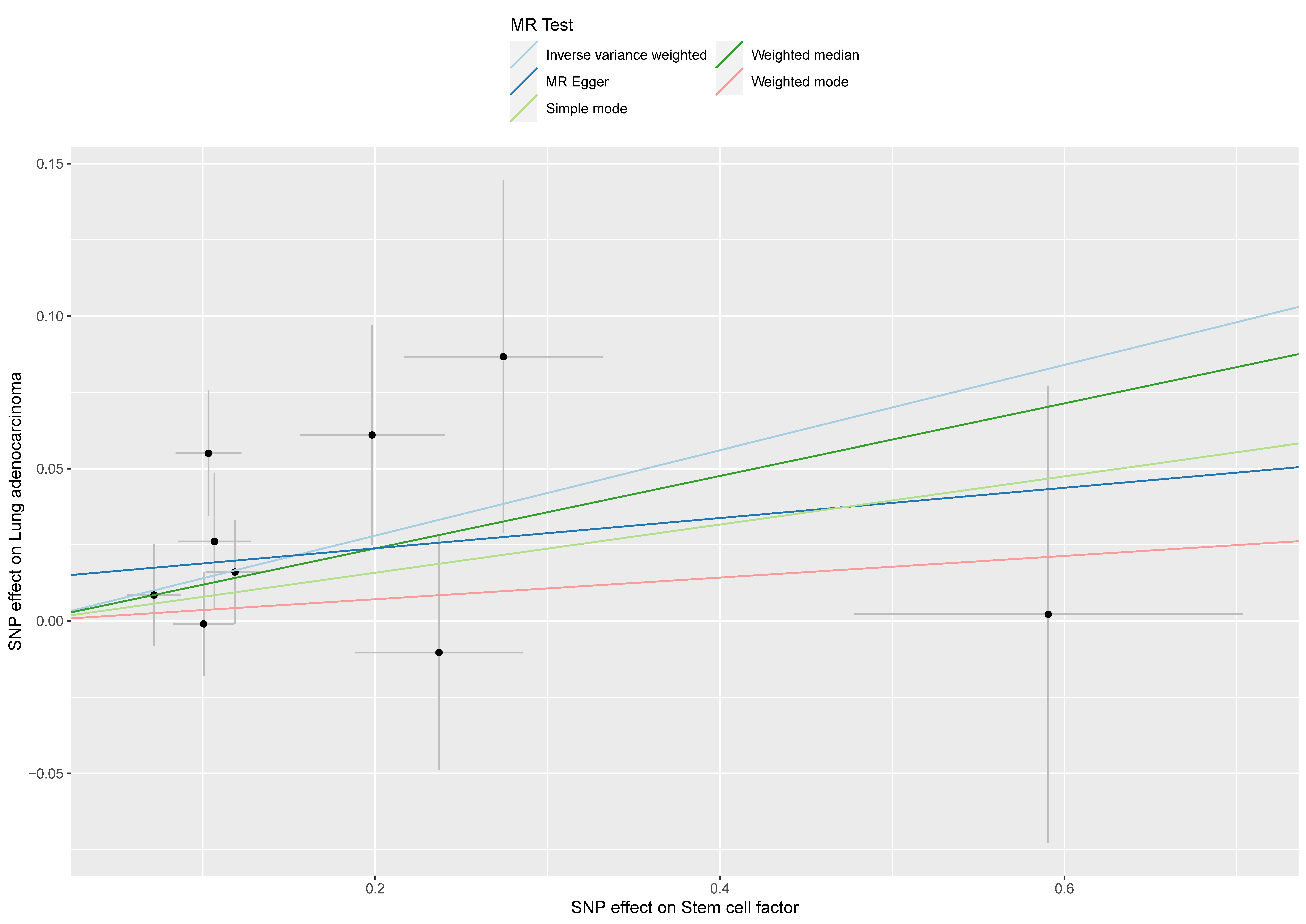

Supplement: Supplementary Figure 2 — Scatter plot of SCF levels on lung lung adenocarcinoma. [file Image_2.tiff]

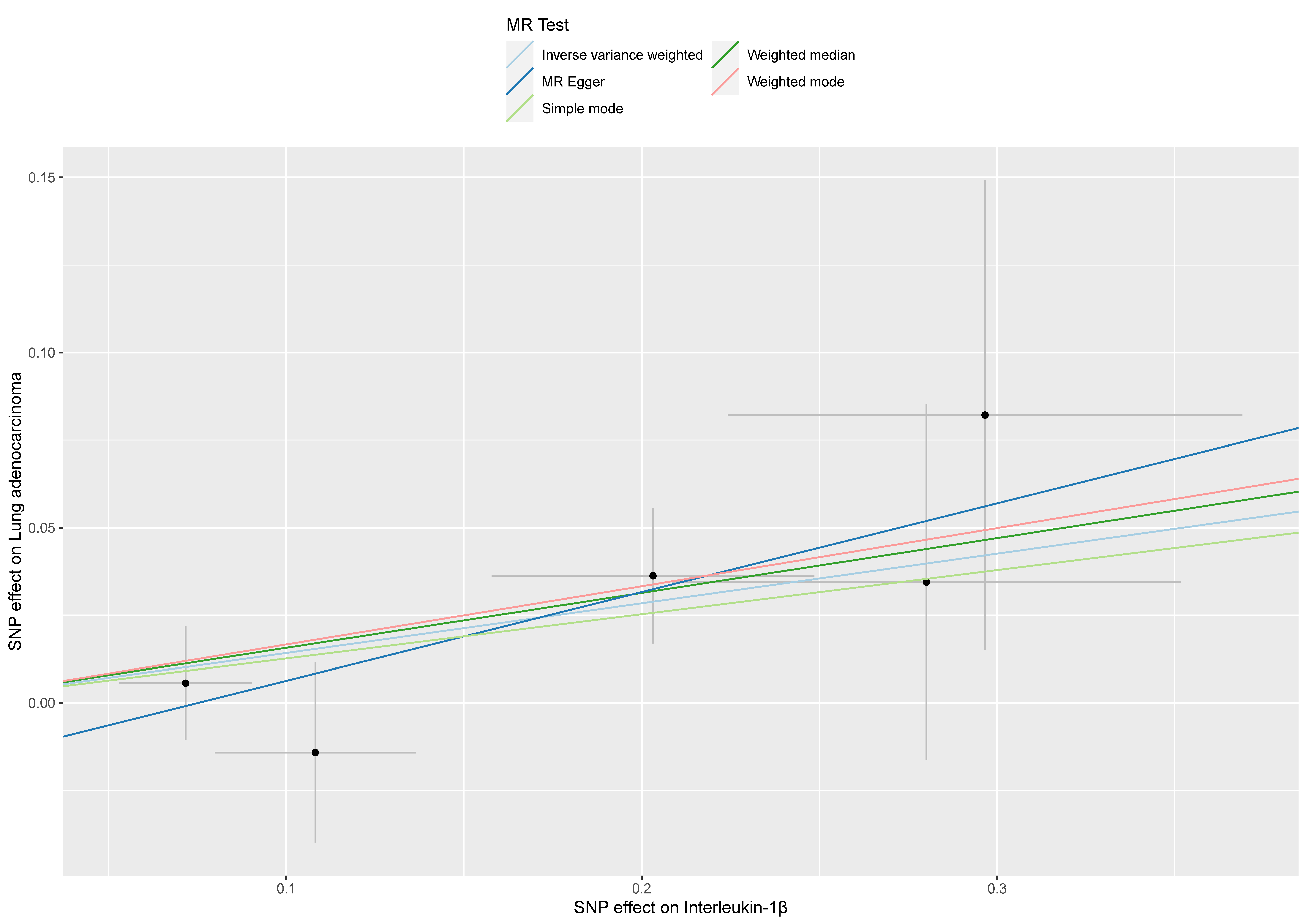

Supplement: Supplementary Figure 3 — Scatter plot of IL-1β levels on lung lung adenocarcinoma. [file Image_3.tiff]

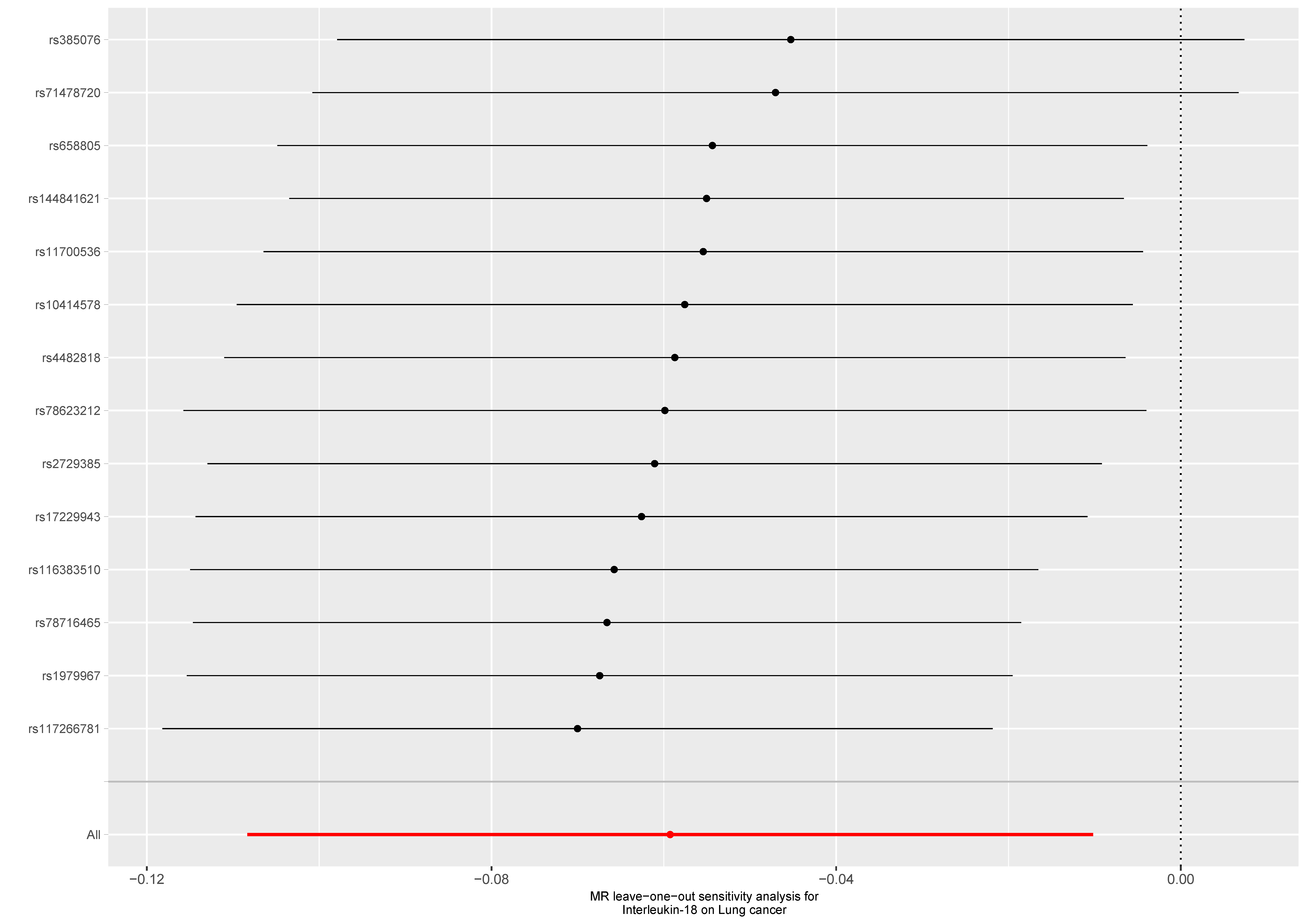

Supplement: Supplementary Figure 4 — Leave-one-out plot of IL-1β levels on lung cancer. [file Image_4.tiff]

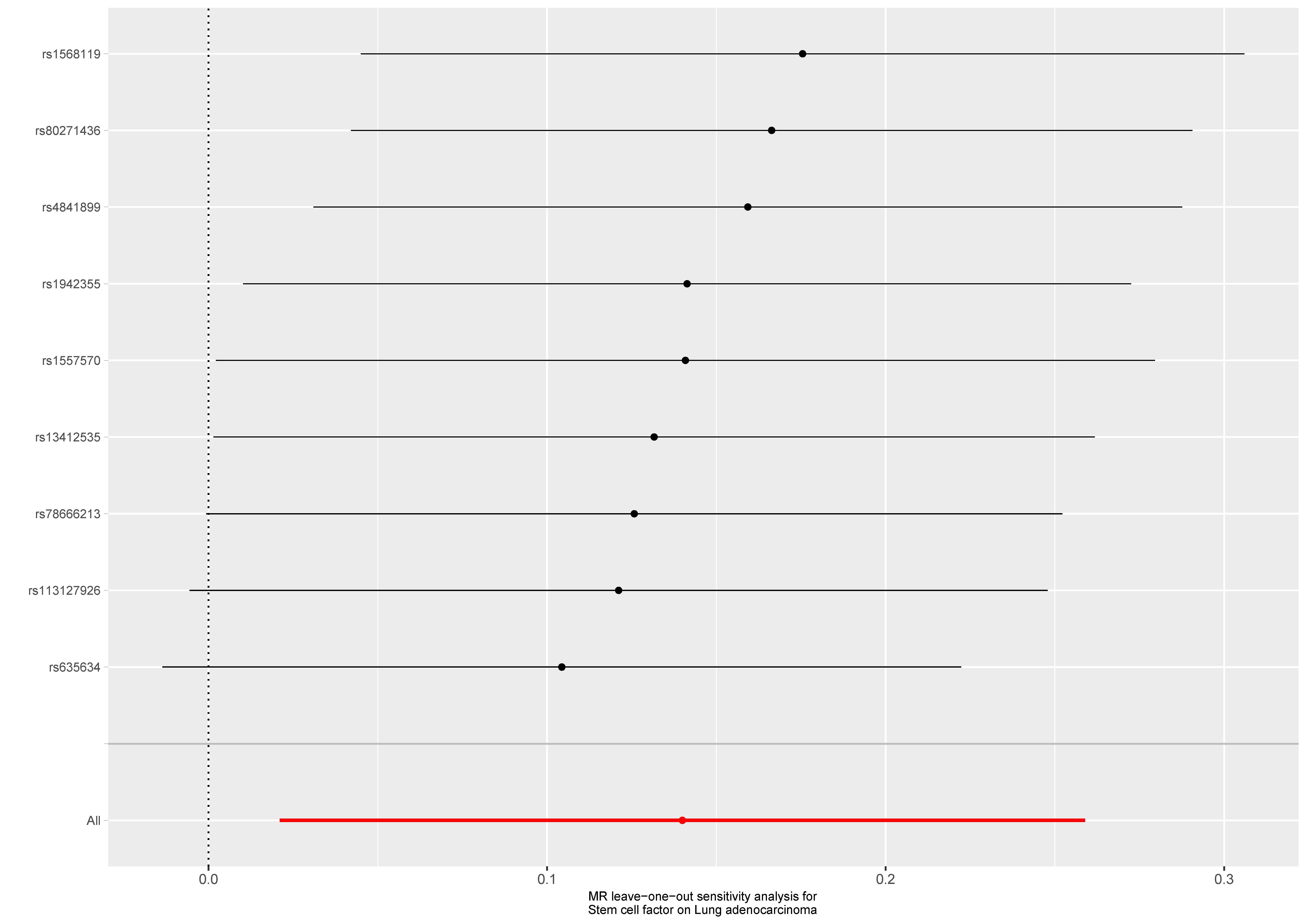

Supplement: Supplementary Figure 5 — Leave-one-out plot of SCF levels on lung adenocarcinoma. [file Image_5.tiff]

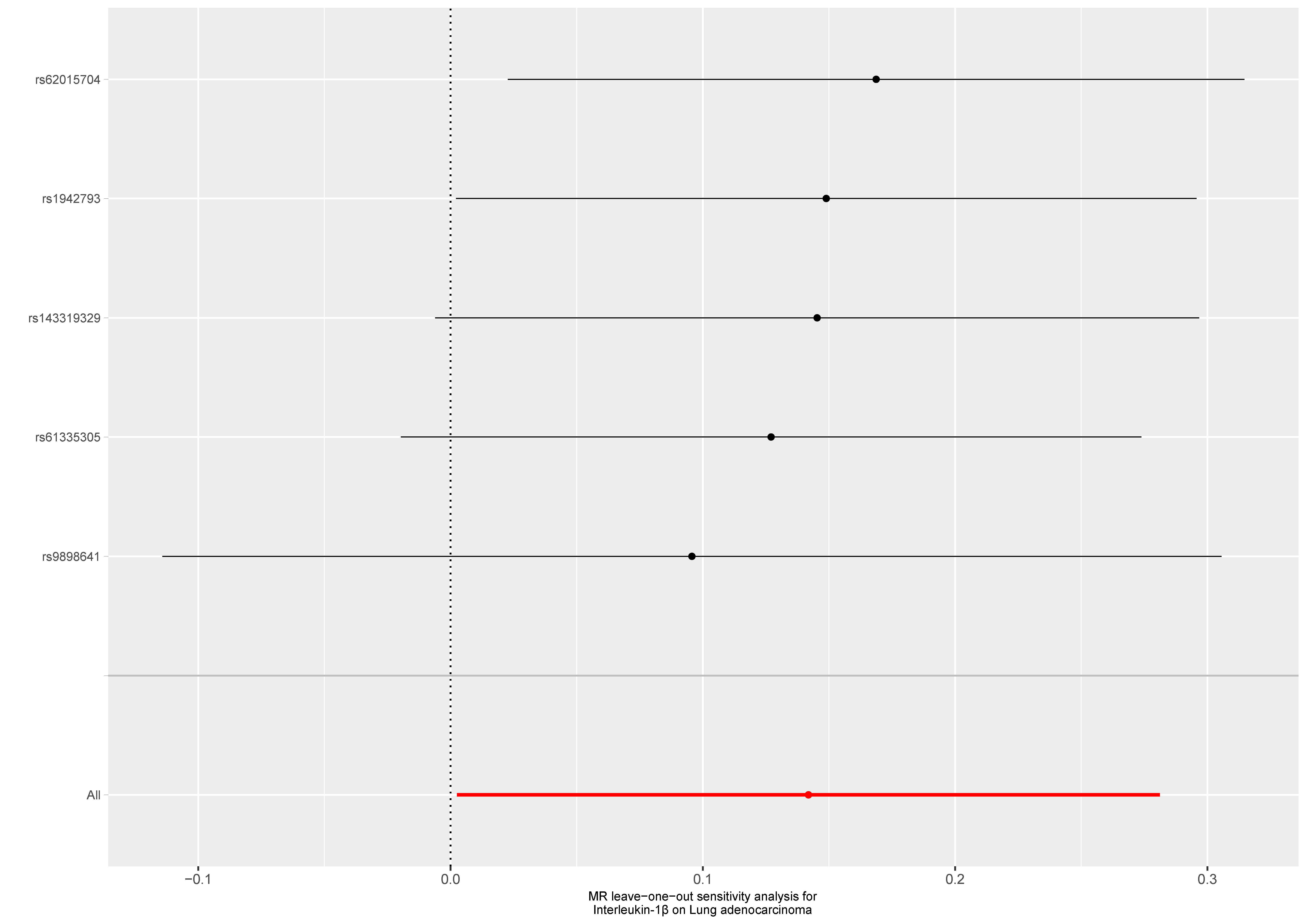

Supplement: Supplementary Figure 6 — Leave-one-out plot of IL-1β levels on lung adenocarcinoma. [file Image_6.tiff]

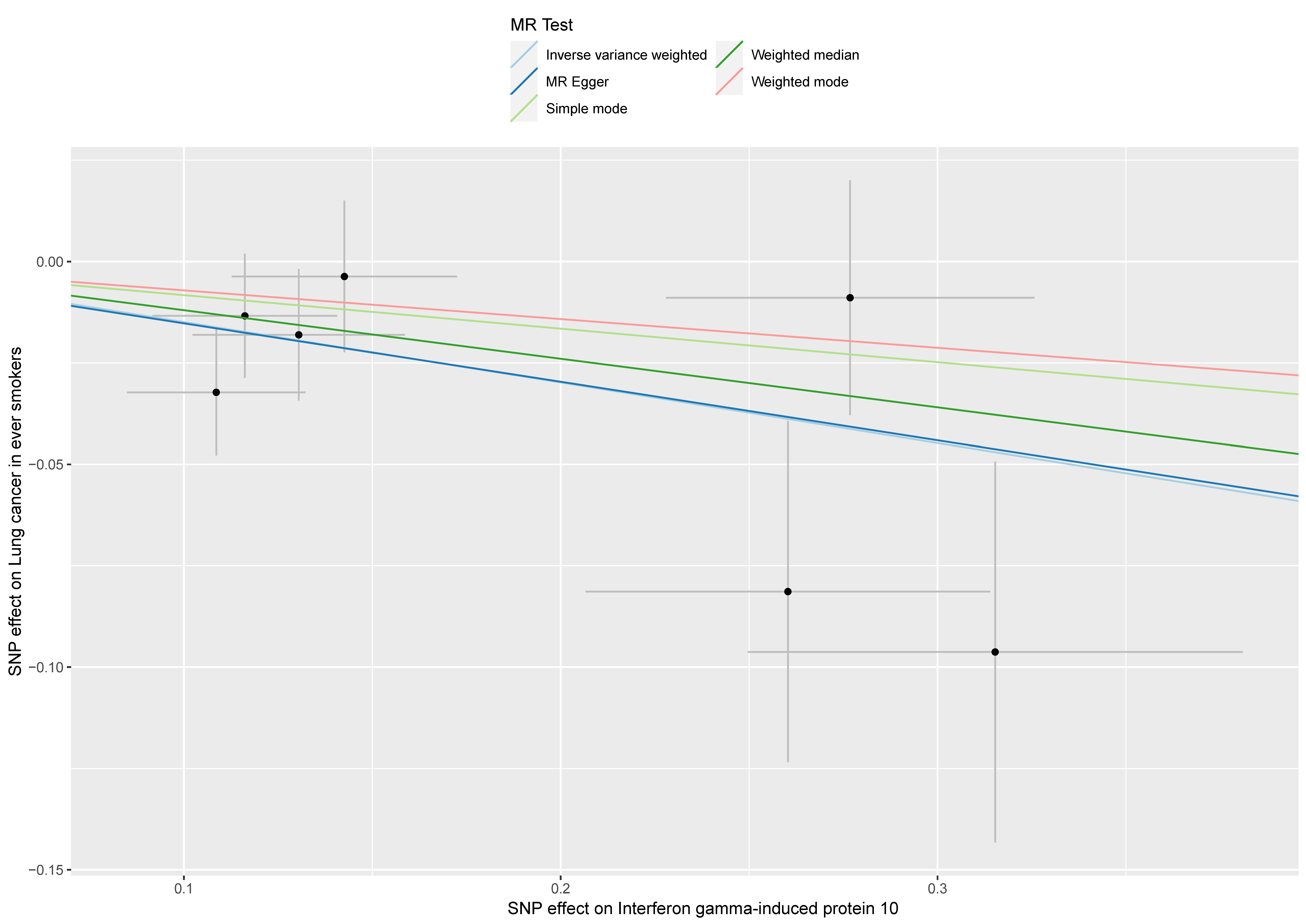

Supplement: Supplementary Figure 7 — Scatter plot of IP-10 levels on lung cancer in ever smokers. [file Image_7.tif]

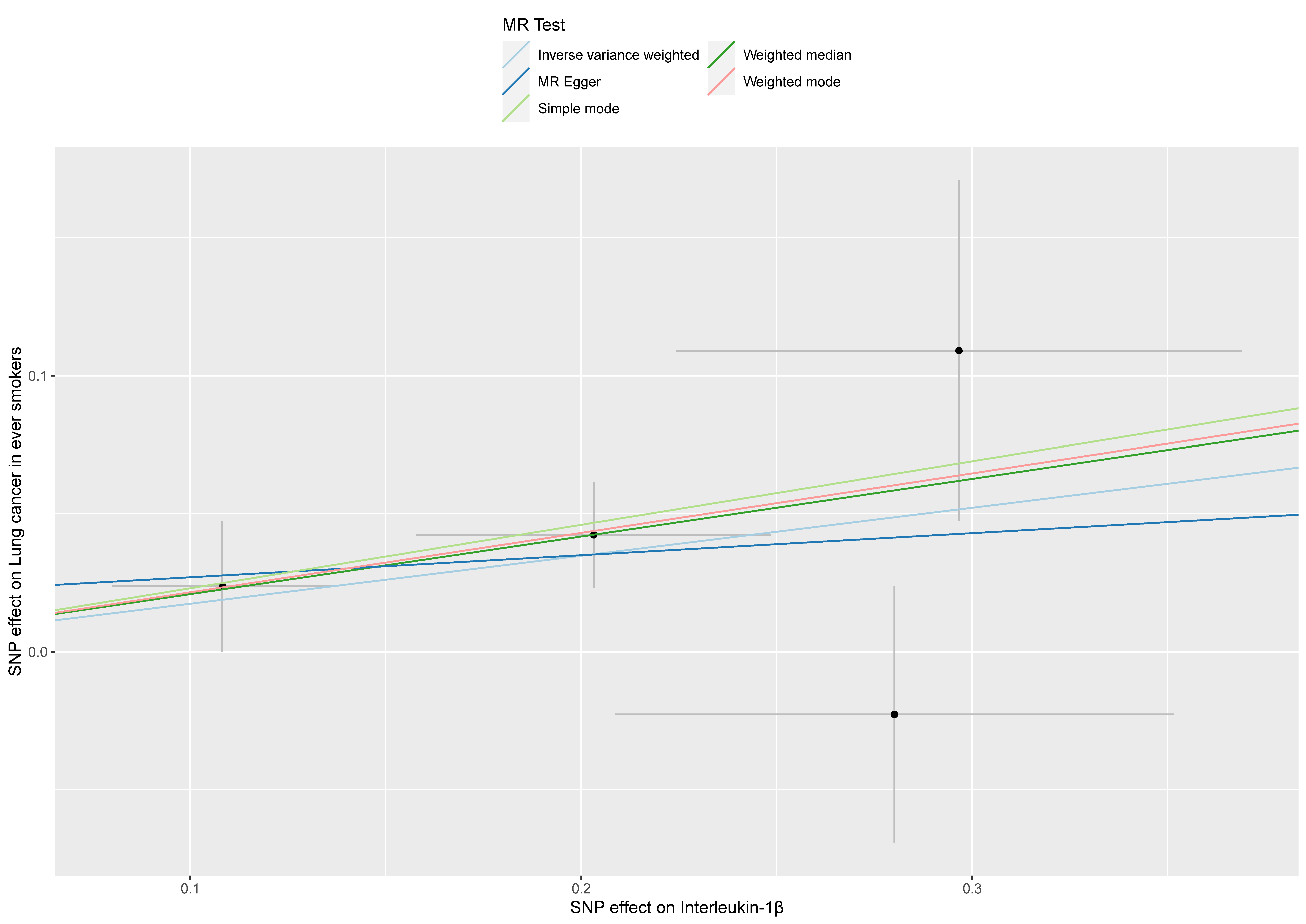

Supplement: Supplementary Figure 8 — Scatter plot of IL-1β levels on lung cancer in ever smokers. [file Image_8.tif]

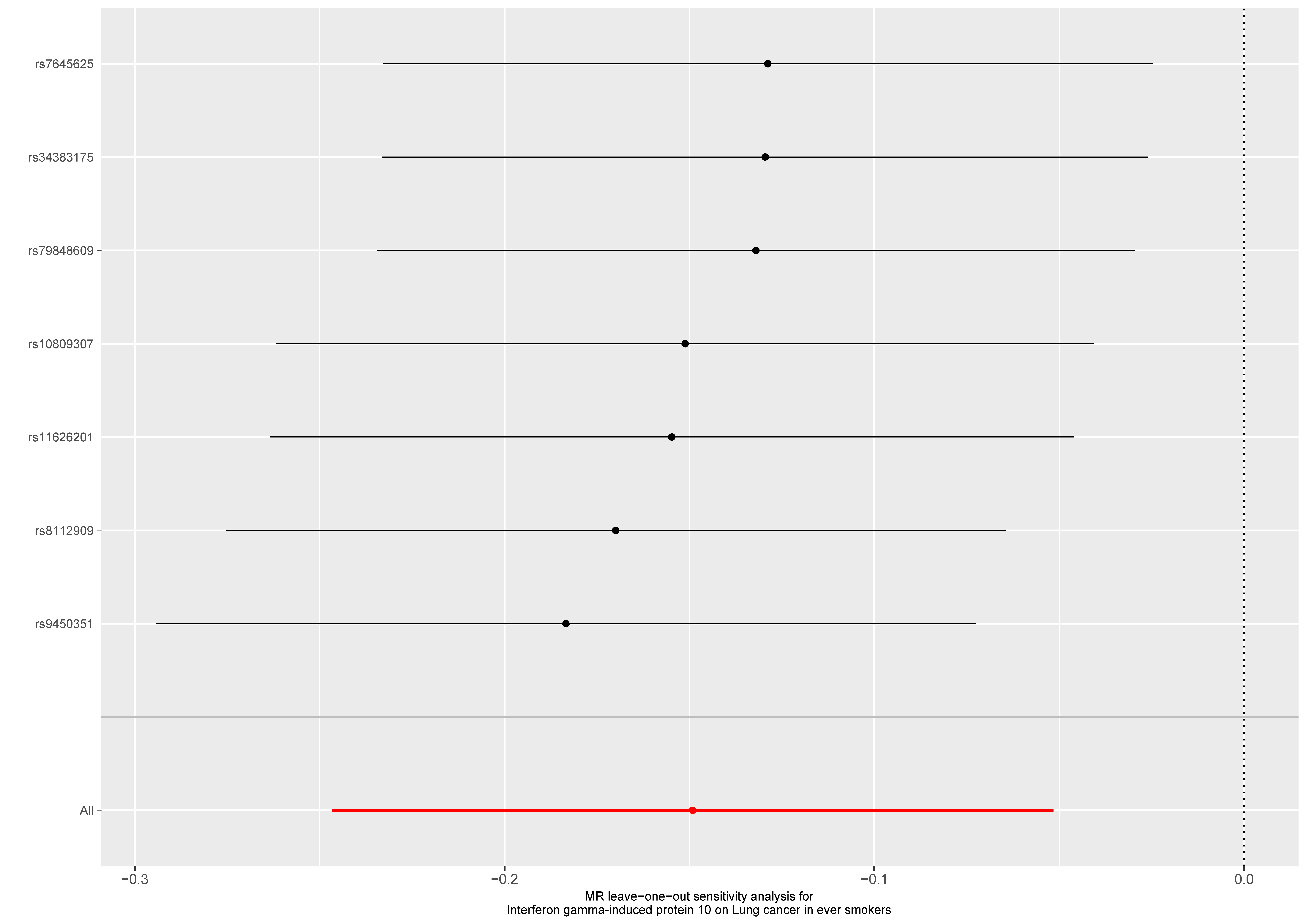

Supplement: Supplementary Figure 9 — Leave-one-out plot of IP-10 levels on lung cancer in ever smokers. [file Image_9.tiff]

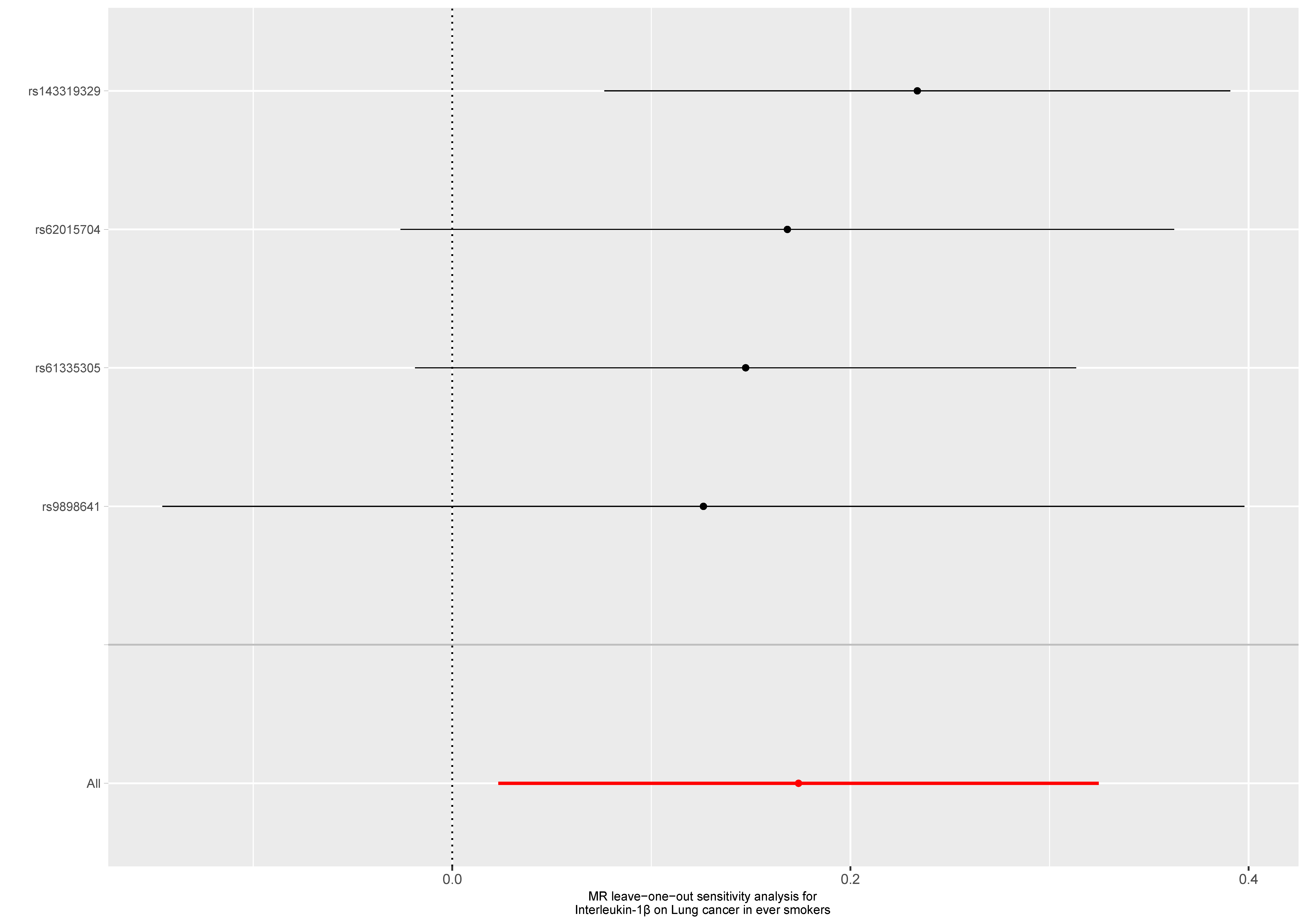

Supplement: Supplementary Figure 10 — Leave-one-out plot of IL-1β levels on lung cancer in ever smokers. [file Image_10.tiff]

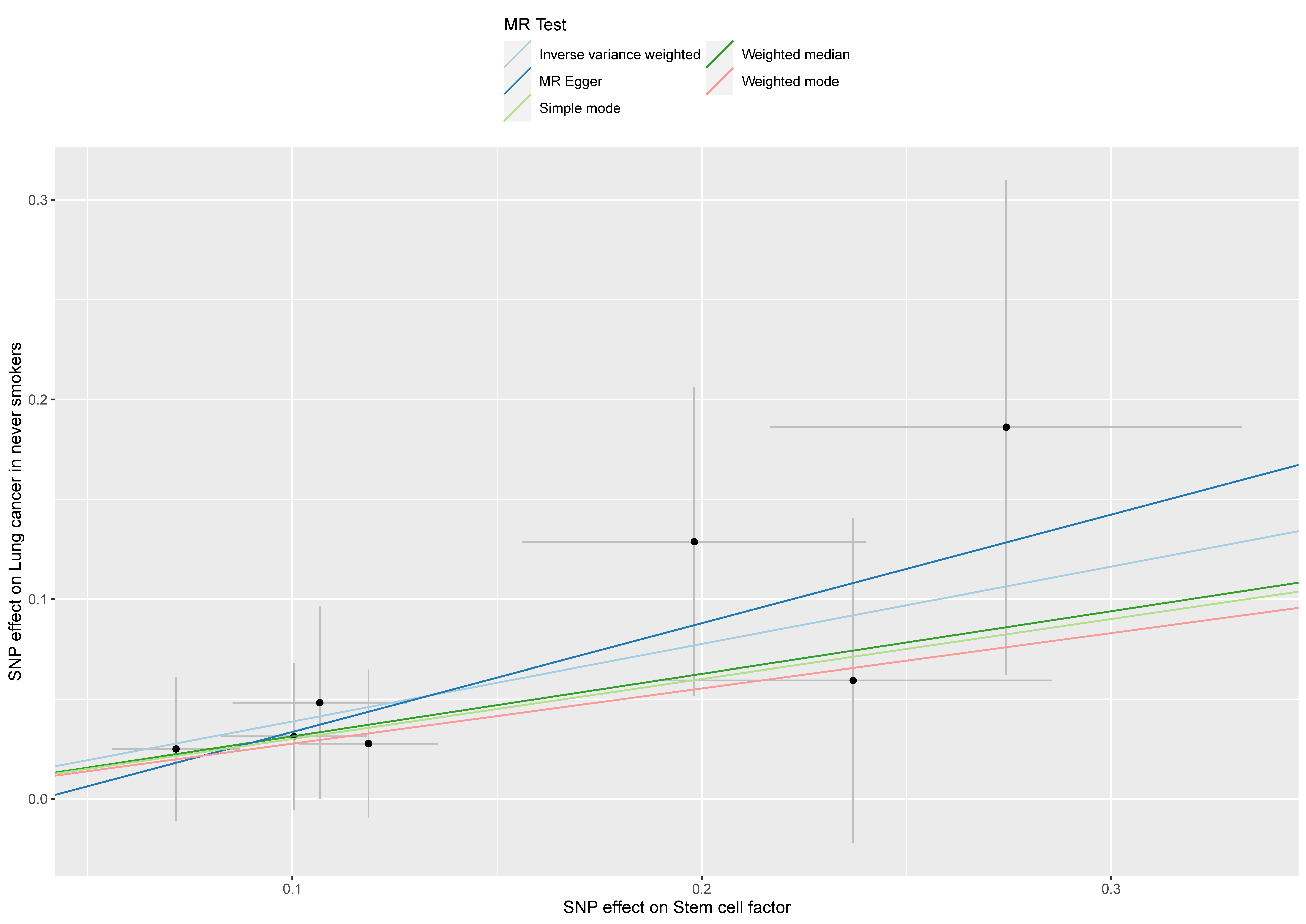

Supplement: Supplementary Figure 11 — Scatter plot of SCF levels on lung cancer in never smokers. [file Image_11.tiff]

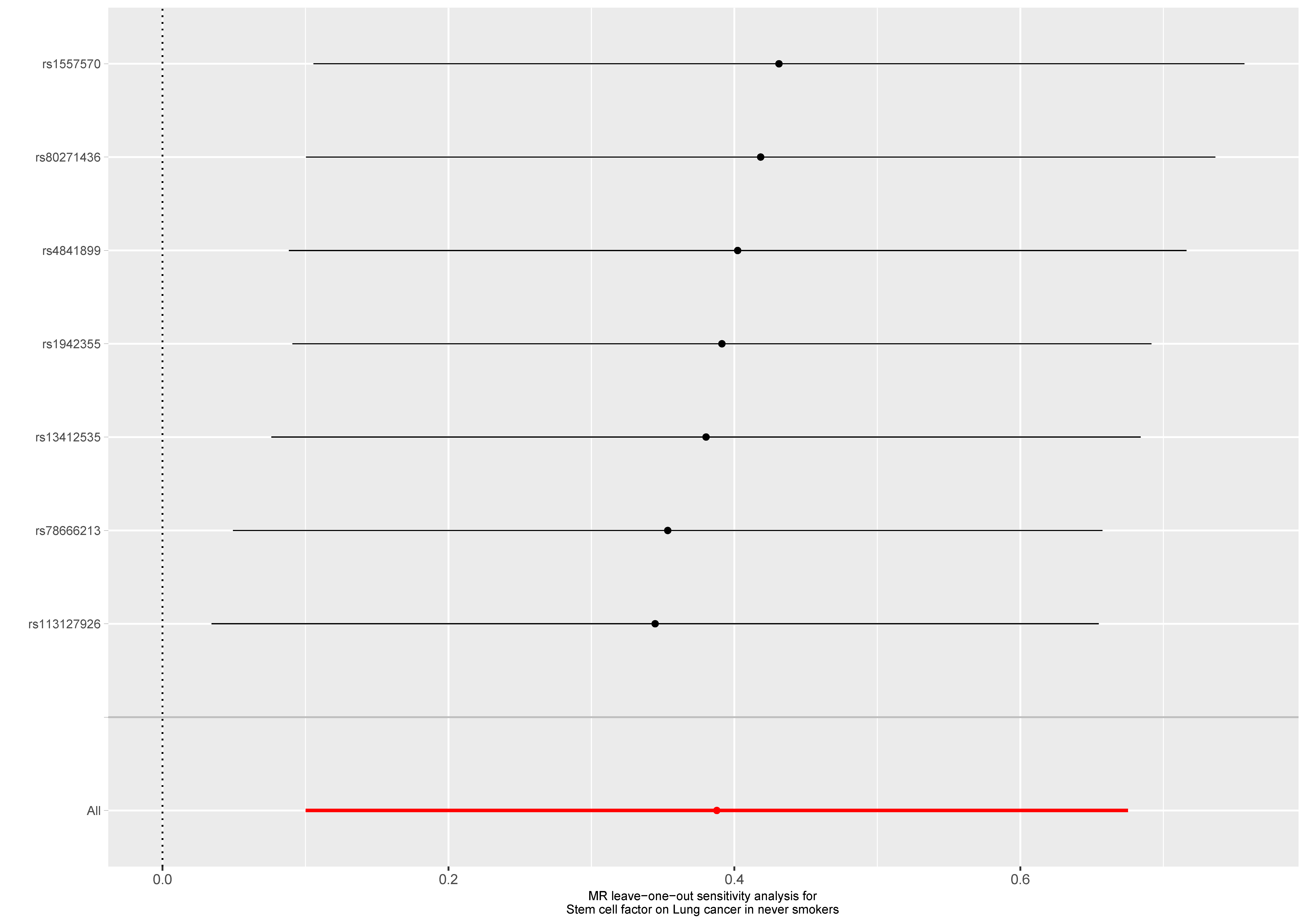

Supplement: Supplementary Figure 12 — Leave-one-out plot of SCF levels on lung cancer in never smokers. [file Image_12.tiff]
